# Immunometabolic Rewiring of Dendritic Cells to Overcome Glutamine‐Driven Immune Suppression in Colorectal Cancer

**DOI:** 10.1002/advs.202513986

**Published:** 2025-10-31

**Authors:** Bingjie Zhang, Renming Fan, Yongrui Hai, Ye Chen, Xintong Lu, Wenhui Wang, Jiarui Dou, Jiaxin Yan, Chang Su, Yue Chen, Le Yang, Minggao Zhao, Lei Liang, Gaofei Wei

**Affiliations:** ^1^ Laboratory of Cellular Metabolism and Precision Therapeutics Institute of Medical Research Northwestern Polytechnical University Xi'an 710072 China; ^2^ Research & Development Institute of Northwestern Polytechnical University in Shenzhen Shenzhen 518057 China; ^3^ Department of Pharmacy Tangdu Hospital The Fourth Military Medical University Xi'an 710038 China; ^4^ Department of Colorectal Surgery Fudan University Shanghai Cancer Center Shanghai 200032 China

**Keywords:** colorectal cancer, dendritic cells, extracellular vesicles, glutamine, immunotherapy, STING

## Abstract

The immunosuppressive tumor microenvironment imposes significant metabolic constraints that impair dendritic cell (DC) maturation and antigen presentation, ultimately undermining antitumor immunity. In colorectal cancer (CRC), elevated glutamine uptake by tumor cells depletes extracellular glutamine, thereby limiting DC functionality and disrupting T cell priming. While glutamine antagonists such as JHU083 inhibit tumor metabolism, they are insufficient to fully restore DC activity. Here, the development of T26, a bifunctional immunometabolic prodrug that links JHU083 with the STING agonist MSA‐2 via a cleavable amide bond, is reported, enabling synchronized intratumoral release and dual targeting of glutamine metabolism and innate immune activation. In murine CRC models, T26 restores DC maturation, promotes CD8⁺ T cell activation, and reprograms tumor cell‐derived extracellular vesicles to enhance antigen presentation and immune stimulation. Importantly, T26 significantly inhibits the growth and proliferation of CRC patient‐derived organoids, underscoring its translational potential in human CRC. Notably, T26 also demonstrates strong synergy with chemotherapy, immune checkpoint blockade, and anti‐angiogenic therapy, significantly improving tumor control without inducing systemic toxicity. These findings position T26 as a mechanistically integrated and translationally promising strategy to overcome glutamine‐driven immune suppression and enhance immunotherapy efficacy in CRC and other metabolically dysregulated malignancies.

## Graphical abstract

T26 is an immunometabolic prodrug that modulates colorectal cancer cells. It blocks glutamine metabolism and enhances STING signaling, which promotes the release of reprogrammed extracellular vesicles. These vesicles drive dendritic cell maturation and CD8⁺ T cell activation, thereby strengthening antitumor immunity and improving the effectiveness of existing therapies.

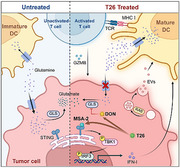

## 1 Introduction

Dendritic cells (DCs) are essential mediators of antitumor immunity, functioning as the primary antigen‐presenting cells that mediate the link between innate and adaptive immune response.^[^ ^1^ ^]^ By capturing tumor‐associated antigens and presenting them on MHC‐I molecules, DCs activate CD8^+^ cytotoxic T lymphocytes, initiating robust immune responses that are essential for tumor eradication.^[^ ^2^ ^]^ However, within the tumor microenvironment (TME), DC maturation is often impaired due to metabolic competition and immunosuppressive signals, resulting in defective antigen presentation and ineffective T‐cell priming.^[^ ^3^ ^]^ Such dysfunction is a major driver of immune evasion and is associated with poor clinical outcomes, particularly in colorectal cancer (CRC).^[^ ^4^, ^5^ ^]^ Enhancing DC maturation and function is therefore a critical strategy for improving the efficacy of immunotherapies, such as immune checkpoint blockade (ICB).^[^ ^6^, ^7^, ^8^ ^]^

Glutamine, a key nutrient for cellular metabolism, plays a central role in DC activation and maturation.^[^ ^9^ ^]^ It supports mitochondrial function, redox balance, and biosynthetic processes essential for immune cell function.^[^ ^10^, ^11^, ^12^ ^]^ However, within the TME, tumor cells exhibit elevated glutamine uptake to support their rapid growth, leading to glutamine depletion in the surrounding extracellular space.^[^ ^13^ ^]^ This depletion restricts glutamine availability for immune cells, particularly DCs, impairing their maturation and antigen‐presenting capacity. As a result, T‐cell activation is compromised, contributing to immune suppression within the TME.^[^ ^9^ ^]^

Targeting glutamine metabolism using pharmacological agents has emerged as a promising therapeutic strategy to combat tumor growth.^[^ ^14^, ^15^ ^]^ 6‐Diazo‐5‐oxo‐l‐norleucine (DON), a potent glutamine antagonist, has shown efficacy in suppressing tumor cell proliferation and enhancing antitumor immunity. However, the clinical application of DON is limited by dose‐limiting gastrointestinal toxicity.^[^ ^16^, ^17^ ^]^ The development of JHU083, a prodrug of DON, provides a more targeted approach by selectively activating the drug within the tumor tissue, thereby reducing systemic toxicity.^[^ ^18^ ^]^ While JHU083 has demonstrated the ability to remodel the TME and promote T‐cell infiltration, it remains insufficient in fully restoring DC function. This suggests that metabolic reprogramming alone is inadequate to fully restore DC function within the TME, highlighting the need for complementary immunostimulatory strategies.

The stimulator of interferon genes (STING) pathway has emerged as a key regulator of innate immunity, particularly in DCs.^[^ ^19^, ^20^ ^]^ Upon activation, STING triggers the production of type I interferons (IFN‐I) and proinflammatory cytokines, promoting DC maturation and enhancing antigen cross‐presentation.^[^ ^21^ ^]^ STING activation also boosts CD8^+^ T cell priming, making it an attractive target for cancer immunotherapy.^[^ ^22^ ^]^ However, STING signaling can be compromised in glutamine‐deprived environments, limiting its efficacy. Based on this, we hypothesized that combining STING activation with glutamine antagonism could synergistically overcome the immunosuppressive effects of glutamine depletion, restore DC function, and enhance antitumor immunity.

To test this hypothesis, we designed T26, a novel bifunctional immunometabolic agent that links JHU083 with MSA‐2, a STING agonist, via a cleavable amide bond.^[^ ^23^ ^]^ This design allows for the synchronized delivery of glutamine antagonism and STING activation directly within the TME. Using murine CRC models, we demonstrated that T26 not only inhibits tumor cell proliferation but also remodels the metabolic landscape, activating innate immune signaling, promoting DC maturation, and enhancing CD8^+^ T cell infiltration and cytotoxic function. Additionally, we found that T26 reprograms tumor‐derived extracellular vesicles (EVs) to enhance antigen presentation and immune activation, providing a novel mechanism of intercellular communication that drives immune responses. Importantly, T26 also exhibited significant synergy with chemotherapies, immunotherapies, and targeted therapies, enhancing tumor control without inducing systemic toxicity.

This study presents a dual‐target approach that combines glutamine antagonism with STING pathway activation to restore DC function. In both CRC murine models and patient‐derived organoids (PDOs), T26 exhibited potent antitumor activity, underscoring its translational potential for CRC therapy. By promoting DC maturation and enhancing T‐cell activation, this strategy improves antitumor immunity while addressing the metabolic obstacles that limit immune responses in CRC. Notably, T26 demonstrates potential in overcoming resistance to existing immunotherapies, offering a way to boost the effectiveness of both chemotherapy and immunotherapy in CRC. These results support the potential of immunometabolic therapies as a promising addition to clinical treatments for CRC and other cancers driven by metabolic reprogramming.

## 2 Results

### 2.1 Dual Activation of Dendritic Cells by Glutamine Antagonists and STING Agonists

**Figure 1:**
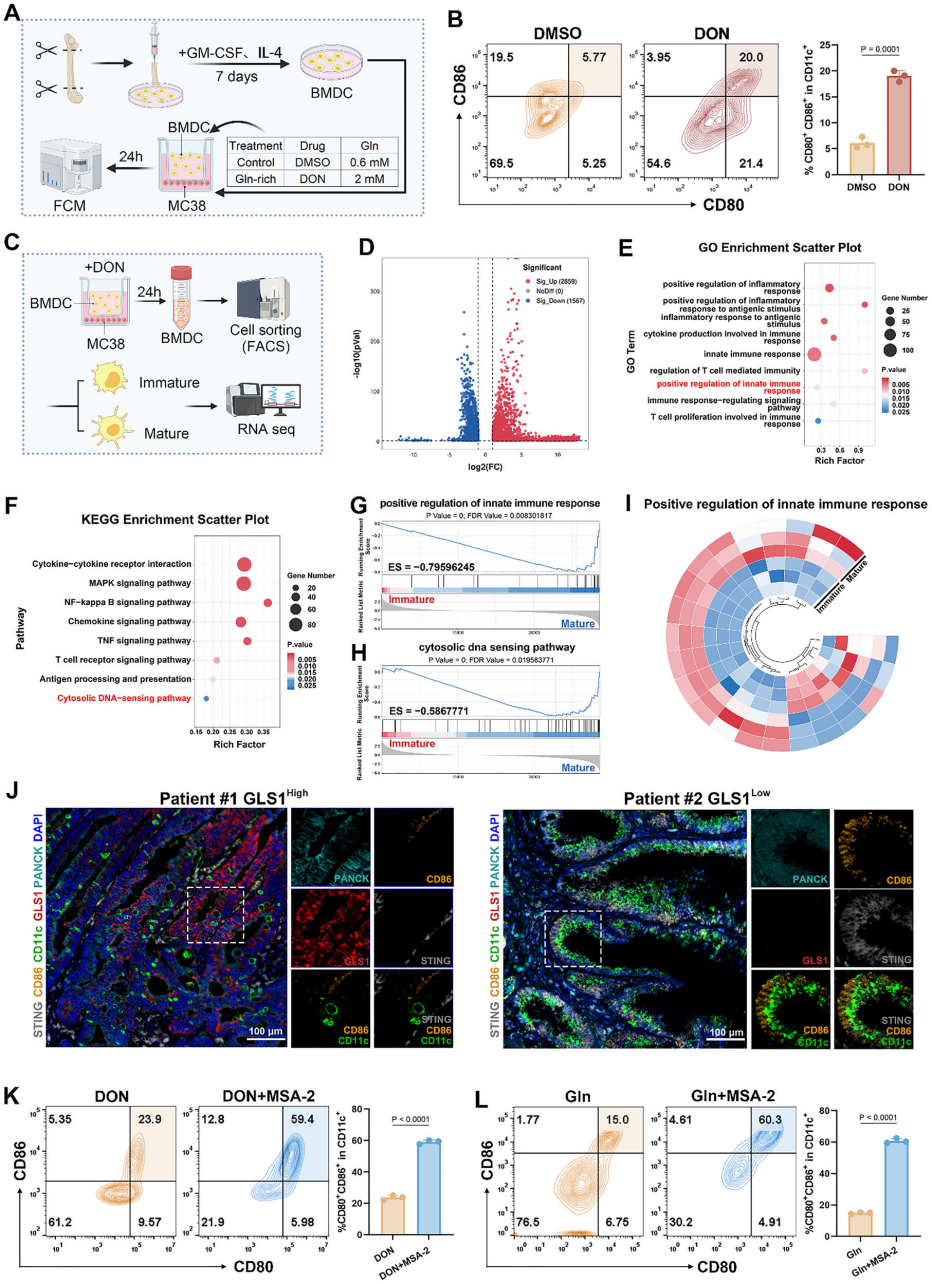
Dual activation of dendritic cells by glutamine antagonists and STING agonists. (A) Schematic representation of the experimental setup. BMDCs were co‐cultured with MC38 tumor cells in a transwell system. (B) Flow cytometry analysis of BMDC maturation in co‐culture with MC38 cells treated with DMSO or DON (5 µm) (*n* = 3). (C) Schematic overview of single‐cell sorting and RNA sequencing analysis of BMDCs cocultured with MC38 cells treated with DMSO or DON (5 µm). Following coculture, mature and immature DCs were sorted for downstream analysis. (D) Volcano plot depicting differentially expressed genes between mature and immature DCs. (E) GO enrichment analysis of immune‐related pathways between mature DCs and immature DCs. (F) KEGG pathway enrichment analysis highlighting immune‐related pathways between mature DCs and immature DCs. (G,H) GSEA plots for pathways significantly changed in DCs between mature and immature groups. (I) Circle heatmap depicting differential expression of genes of the Positive regulation of innate immune response pathway between mature DCs and immature DCs, normalized by z‐score. (J) Multiple immunofluorescence staining for STING expression in two human CRC samples. CD11c represents human dendritic cells. PanCK denotes tumor cells. DAPI indicates nuclei. (K,L) Flow cytometry analysis of BMDCs treated with a combination of MSA‐2 and DON (K) or glutamine (L) (*n* = 3). Error bars represent means ± SD. Differences between groups were tested using one‐way ANOVA followed by Tukey's multiple comparisons test, or unpaired Student's *t*‐test.

Within the TME, cancer cells exhibit heightened glutamine dependency to sustain rapid proliferation and biosynthesis.^[^ ^24^, ^25^ ^]^ This metabolic reprogramming depletes extracellular glutamine, thereby imposing nutrient stress on DCs and leading to impaired maturation and antigen presentation.^[^ ^9^ ^]^ To investigate whether increased extracellular glutamine availability can facilitate DC activation, we first differentiated murine bone marrow cells into bone marrow‐derived dendritic cells (BMDCs) using GM‐CSF and IL‐4 over a 7‐day period. BMDCs were then transferred to a Transwell system and co‐cultured with MC38 cells under different metabolic conditions (**Figure** **1**A).

Two parallel strategies were employed to mimic distinct glutamine states within the TME. In the “control” group, BMDCs were co‐cultured with MC38 cells in the presence of 0.6 mm glutamine and DMSO, simulating a glutamine‐depleted TME caused by excessive glutamine uptake by tumor cells. In contrast, the “Gln‐rich” group included 2 mm glutamine supplementation and glutamine antagonist DON treatment, to simulate a condition where glutamine remains abundant in the extracellular space. The rationale for using DON is that it blocks glutamine metabolism in tumor cells, preventing them from consuming glutamine. This blockade leads to an accumulation of glutamine in the surrounding environment, making it more available for DCs. Under these conditions, we found that DON treatment significantly enhanced BMDC maturation by 12.89% compared to the DMSO control (Figure 1B). Consistent with DON treatment, supplementation with 2 mm glutamine alone also promoted BMDC maturation (Figure S1A, Supporting Information). These results support the hypothesis that targeting tumor glutamine metabolism not only disrupts tumor growth but also alleviates nutrient competition within the TME, thereby restoring DC immunocompetence.

Despite these effects, we observed that approximately 80% of DCs remained in an immature state following DON exposure, suggesting the existence of additional barriers to full activation. To elucidate the molecular basis of this incomplete maturation, we performed transcriptomic profiling of flow‐sorted mature and immature BMDCs after 24 h of DON treatment (Figure 1C). Differential gene expression analysis revealed 4426 genes altered significantly between the two subsets, with 2859 upregulated and 1567 downregulated in mature DCs (Figure 1D; Figure S1B, Supporting Information). Principal component analysis (PCA) revealed distinct clustering of mature and immature BMDCs, reflecting major transcriptional differences between the two states (Figure S1C, Supporting Information). Gene ontology (GO) enrichment analysis highlighted significant upregulation of immune‐related biological processes, including the positive regulation of innate immune response, positive regulation of inflammatory response, and T cell proliferation involved in immune response (Figure 1E). KEGG pathway enrichment further revealed that key signaling axes relevant to DC functionality were engaged, including the cytosolic DNA sensing pathway, cytokine–cytokine receptor interaction, chemokine signaling pathway, NF‐kappa B signaling pathway, and T cell receptor signaling pathway (Figure 1F).

Gene set enrichment analysis (GSEA) demonstrated that the positive regulation of innate immune response and cytosolic DNAsensing pathway was significantly downregulated in the immature DC population (Figure 1G–I; Figure S1D, Supporting Information), underscoring their impaired immunostimulatory capacity. As professional antigen‐presenting cells, DCs are central to initiating tumor‐specific CD8⁺ T cell responses through cross‐presentation of tumor‐associated antigens.^[^ ^26^ ^]^ One of the key mediators of innate immune activation in DCs is the cyclic GMP‐AMP synthase–stimulator of interferon genes (cGAS–STING) pathway, which senses cytosolic DNA and drives type I interferon production.^[^ ^27^, ^28^, ^29^, ^30^, ^31^ ^]^ Given the central role of STING signaling in DC function and its involvement in the enriched pathways identified above, we next sought to examine the in vivo relevance of this axis in human tumors. To this end, we performed multiplex immunofluorescence staining on clinical colorectal cancer specimens to assess the relationship between glutamine metabolism, DC maturation, and STING expression. In tumor tissues exhibiting high glutamine metabolic activity, we observed significantly decreased STING expression, accompanied by a marked reduction in mature DCs (Figure 1J), suggesting a mechanistic link between glutamine metabolism and STING pathway suppression.

To determine whether pharmacologic activation of STING could rescue DC maturation under glutamine‐restricted conditions, we treated co‐cultures with a combination of DON and the STING agonist MSA‐2. This dual treatment significantly increased the proportion of mature DCs to 59.06%, substantially higher than either agent alone (Figure 1K). Notably, a similar enhancement of DC activation was observed when exogenous glutamine was combined with MSA‐2 (Figure 1L), further supporting the functional convergence of glutamine availability and STING signaling in DC regulation.

Collectively, these findings demonstrate that glutamine antagonism alone only partially restores DC maturation due to concurrent suppression of innate immune response. The combined targeting of tumor glutamine metabolism and innate immune activation through the STING pathway yields synergistic enhancement of DC function.

### 2.2 T26 Modulates Tumor Cells through Glutamine Antagonism and STING Pathway Activation

**Figure 2:**
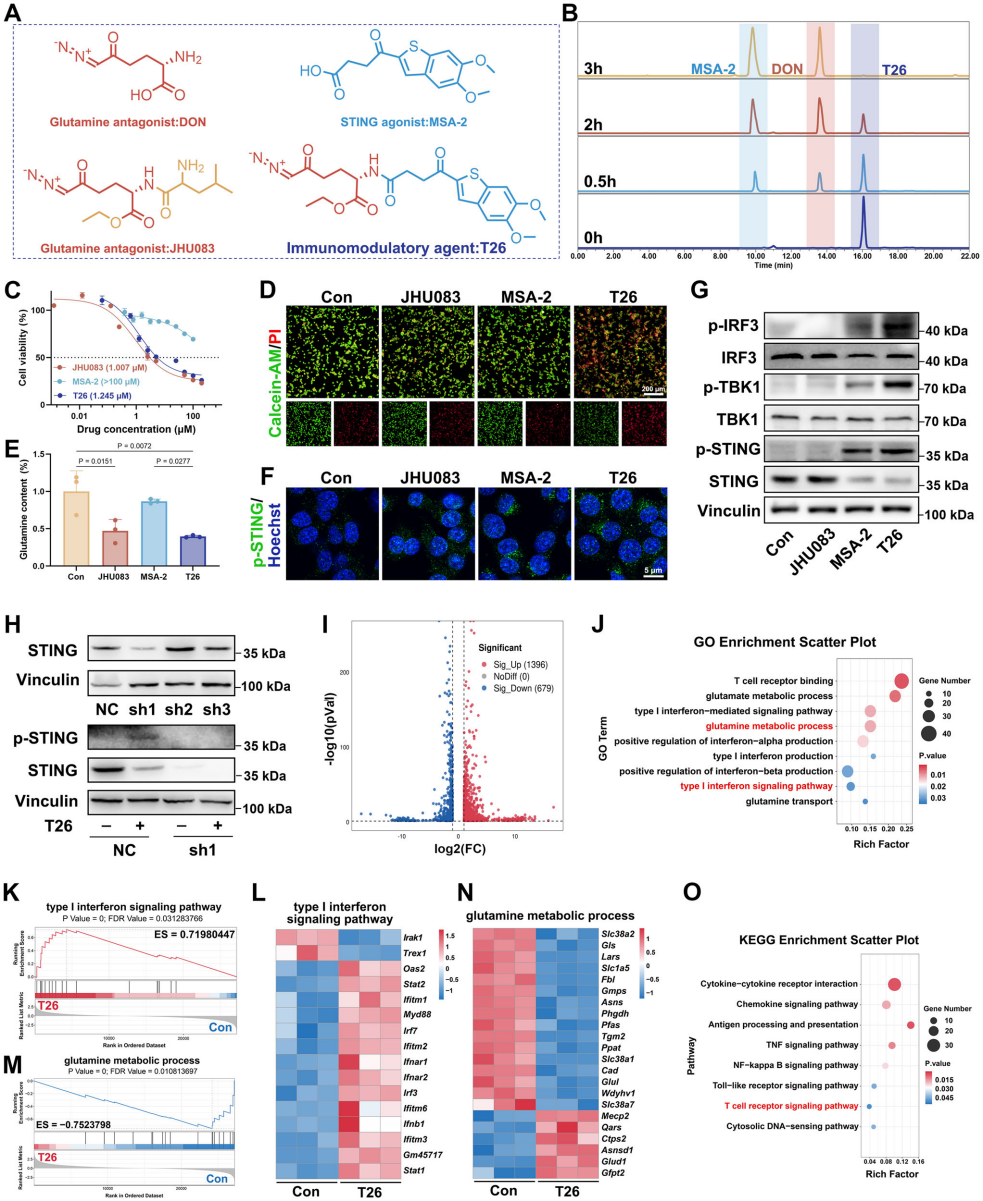
Design, characterization, and functional validation of T26. (A) The chemical structure of T26, where the blue part represents the STING agonist MSA‐2, and the red part represents DON. (B) High‐performance liquid chromatography (HPLC) analysis demonstrating controlled release of T26. (C) MTT assay showing the cytotoxic effects of T26 on MC38 tumor cells (*n* = 3). (D) Immunofluorescence staining of live and dead MC38 cells following T26 treatment. MC38 cells treated with DMSO, JHU083 (5 µm), MSA‐2 (5 µm), or T26 (5 µm) for 24 h. (E) Intracellular glutamine quantification in MC38 upon treated with DMSO, JHU083 (5 µm), MSA‐2 (5 µm), or T26 (5 µm) for 24 h (*n* = 3). (F) IF staining of p‐STING expression in MC38 cells. (G) Western blot analysis of phosphorylated STING, TBK1, and IRF3 in T26‐treated MC38 cells. (H) STING knockdown using siRNA abrogates the effects of T26. (I) Volcano plot depicting differentially expressed genes between T26‐treated and untreated groups. (J) GO enrichment analysis of immune‐related pathways in T26‐treated MC38 cells. (K) GSEA showing significant upregulation of the Positive regulation of type I interferon production pathway upon T26 treatment. (L) Heatmap depicting differential expression of genes of type I interferon production pathway between T26‐treated and untreated groups, normalized by z‐score. (M) Downregulation of the glutamine metabolic process pathway in T26‐treated MC38 cells. (N) Heatmap depicting differential expression of genes of the glutamine metabolic process pathway between T26‐treated and untreated groups, normalized by z‐score. (O) KEGG pathway analysis indicating enrichment of immune‐related pathways. Error bars represent means ± SD. Differences between groups were tested using one‐way ANOVA followed by Tukey's multiple comparisons test, or unpaired Student's *t*‐test.

JHU083, a prodrug of DON, demonstrates improved pharmacokinetic properties and reduced systemic toxicity compared to DON, primarily due to its tumor‐selective activation. JHU083 undergoes enzymatic cleavage of its ester and amide bonds, leading to the localized release of DON within tumor tissues, thereby inhibiting glutamine metabolism.^[^ ^18^ ^]^ Given emerging evidence that STING pathway activation can further enhance the immunostimulatory effects of glutamine antagonists on DCs, we rationally designed a bifunctional immunometabolic agent, T26. In this construct, the leucine moiety of JHU083 was replaced with the STING agonist MSA‐2, while retaining the hydrolyzable amide linkage. This structural modification enables enzyme‐responsive release of both active entities specifically within the TME. The rationale for this conjugation strategy is to ensure spatiotemporally coordinated delivery of JHU083 and MSA‐2 to the tumor site, thereby maximizing intratumoral synergy. The chemical structure and synthetic route of T26 are depicted in **Figure** **2**A and Scheme S1 (Supporting Information), confirmed by ^1^H and ^13^C NMR spectroscopy, as well as high‐resolution mass spectrometry (HRMS) (Figures S2–S4, Supporting Information). High‐performance liquid chromatography (HPLC) demonstrated that T26 is susceptible to esterase‐mediated hydrolysis, enabling controlled and sustained release of DON under physiological conditions (Figure 2B). The pharmacokinetic profile of T26 in the MC38 model demonstrates its capacity for the spatiotemporally coordinated delivery of therapeutic agents (Figure S5A, Supporting Information), as evidenced by a substantially higher systemic exposure in the tumor than in plasma.

To investigate the direct effects of T26 on tumor cells, we treated MC38 murine colon cancer cells with varying concentrations of T26, and cell viability was evaluated using the MTT assay. T26 exhibited potent cytotoxicity toward MC38 cells, with an IC_50_ of 1.245 µm (Figure 2C). Consistent with this finding, immunofluorescence staining of live and dead cells confirmed increased tumor cell death upon T26 treatment (Figure 2D). Beyond the direct cytotoxic effects, T26 also demonstrated a superior capacity to suppress tumor metastasis by significantly inhibiting the migration and invasion of MC38 cells, outperforming both MSA‐2 and JHU083 alone (Figure S5B,C, Supporting Information). Furthermore, MTT assay results demonstrated that T26 treatment did not impair the viability of DC2.4 cells at the tested dosage (Figure S5D, Supporting Information). We also measured intracellular and extracellular glutamine levels in MC38 cells following 24 h of T26 exposure. The results showed a significant reduction in intracellular glutamine content compared to the control group, along with an increase in extracellular glutamine levels (Figure 2E; Figure S5E, Supporting Information), confirming that T26 effectively antagonizes glutamine metabolism in tumor cells. Importantly, these findings also indicate that the incorporation of the STING agonist MSA‐2 does not impair the glutamine‐depleting function of the prodrug.

Next, we examined whether T26 could activate STING signaling in tumor cells. Immunofluorescence analysis revealed a marked increase in phosphorylated STING (p‐STING) and phosphorylated interferon regulatory factor 3 (p ‐ IRF3) following T26 treatment (Figure 2F; Figure S5F, Supporting Information). Western blot analysis further demonstrated enhanced phosphorylation of the canonical STING downstream effectors TANK‐binding kinase 1 (TBK1) and IRF3, consistent with robust pathway activation (Figure 2G). To verify that these effects were STING‐dependent, we performed siRNA‐mediated knockdown of STING in MC38 cells. In STING‐deficient cells, T26‐induced p‐STING expression was significantly attenuated (Figure 2H), reinforcing the conclusion that T26 exerts its immunostimulatory effects through STING pathway activation.

To further elucidate the molecular mechanisms underlying T26's effects, we conducted RNA sequencing of MC38 cells treated with T26. Transcriptomic analysis revealed 2075 significantly differentially expressed genes, with 1396 upregulated and 679 downregulated (Figure 2I; Figure S5G, Supporting Information). PCA revealed distinct clustering between control and T26‐treated MC38 cells, indicating substantial transcriptional differences between the two conditions (Figure S5H, Supporting Information). GO enrichment analysis identified several immune‐related biological processes, including positive regulation of type I interferon production, glutamine metabolic process, and glutamine transport (Figure 2J). GSEA confirmed significant upregulation of the type I interferon response pathway, consistent with activation of the cGAS–STING signaling axis by T26 (Figure 2K,L). In parallel, glutamine metabolism pathways were downregulated following T26 treatment (Figure 2M), including marked suppression of *Slc38a2* (encoding glutamine transporter) and *Gls* (encoding glutaminase) (Figure 2N), further corroborating the glutamine antagonism function of T26. Additionally, KEGG pathway analysis revealed significant enrichment of the T cell receptor signaling pathway (Figure 2O), suggesting that T26 may enhance the immunogenicity of tumor cells and facilitate their recognition by cytotoxic T lymphocytes. Consistently, GSEA demonstrated significant upregulation of this pathway, further supporting the immunostimulatory potential of T26 (Figure S5I, Supporting Information).

In summary, T26 exerts a robust antitumor effect in vitro through a dual mechanism involving STING pathway activation and glutamine metabolism inhibition. These findings support the potential of T26 as a rationally engineered immunometabolic modulator capable of remodeling the tumor microenvironment and enhancing antitumor immune responses.

### 2.3 Oral Administration of T26 Elicits Potent and Safe Antitumor Responses in Murine Colorectal Cancer Models

**Figure 3:**
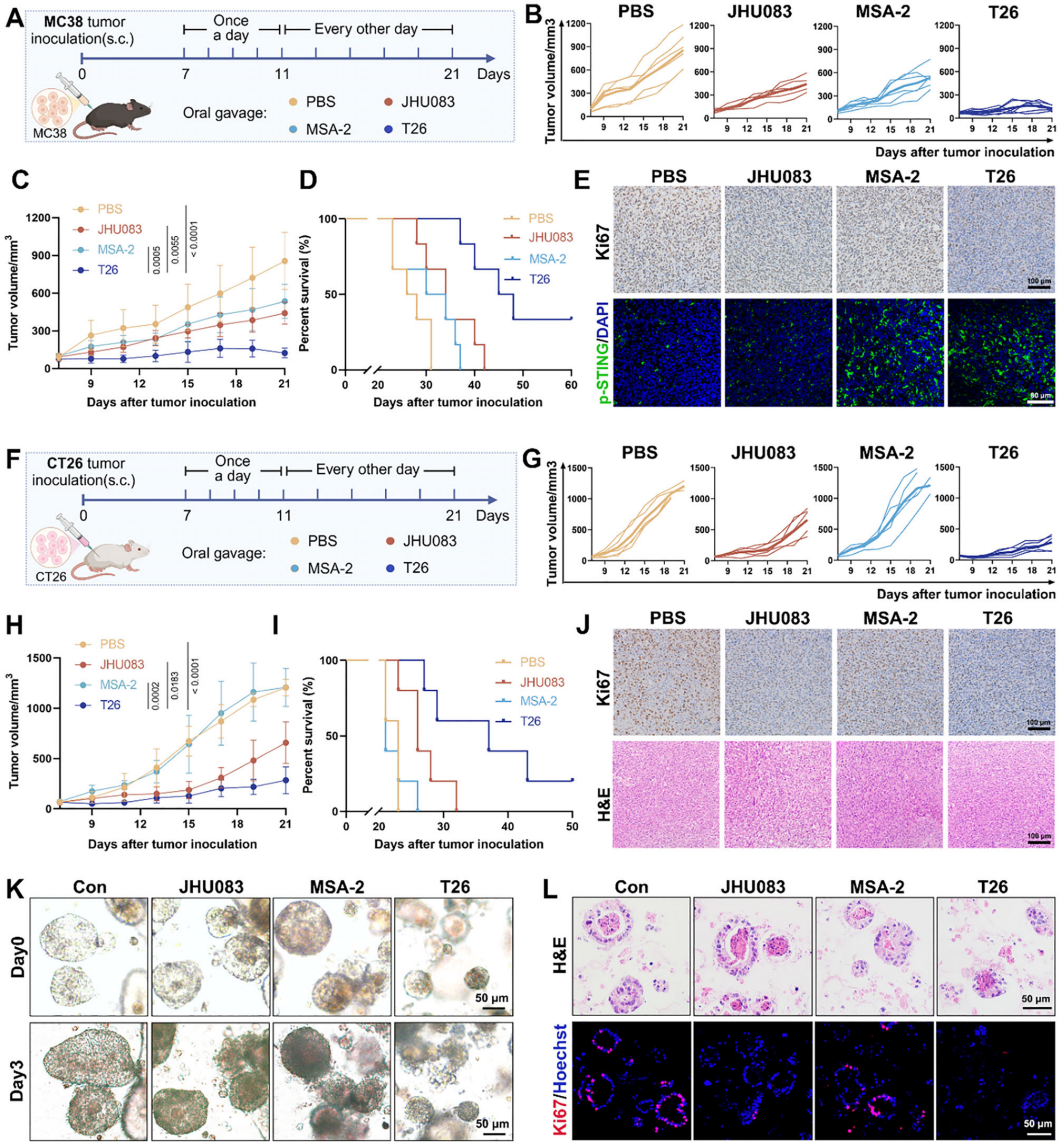
Oral administration of T26 suppresses tumor growth in colorectal cancer models. (A) The dosing schedule: C57BL/6 mice were inoculated with MC38 cells and treated with PBS, mice were randomly assigned to receive either JHU083 or MSA‐2 at 1 mg kg^−1^ day^−1^ from day 7 to 11, followed by 0.3 mg kg^−1^ every other day from day 12 to 21, or T26 at 1.5 mg kg^−1^ day^−1^ from day 7 to 11, followed by 0.45 mg kg^−1^ every other day from day 12 to 21. (B,C) Tumor growth of MC38 tumors in C57BL/6 mice with different treatment (*n* = 6). (D) Kaplan–Meier survival analysis of MC38‐bearing mice with different treatment (*n* = 6). (E) IHC analysis of Ki67 and IF staining of p‐STING expression in MC38 tumors. (F) Experimental design: BALB/C mice were inoculated with CT26 cells, mice were randomly assigned to receive either JHU083 or MSA‐2 at 1 mg kg^−1^ day^−1^ from day 7 to 11, followed by 0.3 mg kg^−1^ every other day from day 12 to 21, or T26 at 1.5 mg kg^−1^ day^−1^ from day 7 to 11, followed by 0.45 mg kg^−1^ every other day from day 12 to 21. (G,H) Tumor growth of CT26 tumors in BALB/C mice with different treatment (*n* = 5). (I) Kaplan–Meier survival analysis of CT26‐bearing mice with different treatment (*n* = 5). (J) Ki67 and H&E staining of CT26 tumors. (K) Bright‐field microscopy images of PDO CRC before and after treatment with DMSO, JHU083 (10 µm), MSA‐2 (10 µm), or T26 (10 µm) for 3 days. (L) Representative H&E staining and Ki67 immunofluorescence staining of CRC PDO following treatment with DMSO, JHU083 (10 µm), MSA‐2 (10 µm), or T26 (10 µm) for 5 days. Error bars represent means ± SD. Differences between groups were tested using one‐way ANOVA followed by Tukey's multiple comparisons test, or unpaired Student's *t*‐test.

To evaluate the therapeutic efficacy and translational potential of T26, we conducted in vivo studies in two well‐established syngeneic mouse models of colorectal cancer: MC38 and CT26. Notably, T26 was administered via oral gavage, a clinically favorable route, highlighting its potential for systemic administration. Mice were treated T26 or equimolar doses of the individual agents JHU083 or MSA‐2 according to a predefined regimen (**Figure** **3**A).

In MC38 tumor‐bearing mice, T26 treatment led to a marked reduction in tumor growth compared to both monotherapies JHU083 and MSA‐2 (Figure 3B,C). More importantly, T26 treatment significantly prolonged overall survival, underscoring its capacity not only to control tumor burden but also to improve clinical outcome (Figure 3D). Histological analysis revealed a pronounced suppression of tumor cell proliferation, evidenced by diminished Ki67 expression and corroborated by H&E staining. Mechanistically, T26 effectively activated the STING pathway in vivo, as indicated by elevated levels of phosphorylated STING and phosphorylated IRF3 (Figure 3E; Figure S6A, Supporting Information), further validating the dual‐functional design of the molecule. Beyond efficacy, we also comprehensively evaluated the safety profile of T26. Histopathological analyses of major organs, including the heart, liver, spleen, lungs, and kidneys, revealed no signs of systemic toxicity such as tissue damage, necrosis, or inflammatory infiltration (Figure S6B, Supporting Information). Furthermore, the absence of significant body weight loss throughout treatment supports the favorable tolerability of T26 (Figure S6C, Supporting Information).

To assess the broader applicability of these findings, we extended the study to the CT26 model (Figure 3F). Consistent with the MC38 results, T26 exhibited superior tumor control compared to either JHU083 or MSA‐2 alone (Figure 3G,H), and significantly prolonged survival (Figure 3I). Tumors from T26‐treated mice showed marked reductions in Ki67 positivity and histological malignancy (Figure 3J), further confirming its antiproliferative efficacy. Meanwhile, the activation of the STING pathway by T26 in the CT26 model was confirmed by the elevated levels of p‐STING and p‐IRF3 (Figure S6D, Supporting Information). No systemic toxicity was observed, as demonstrated by intact histology in non‐tumor tissues and stable body weight (Figure S6E,F, Supporting Information).

To investigate the therapeutic efficacy of T26 against tumor metastasis, we established a pulmonary metastasis model by intravenously injecting MC38 cells into C57BL/6 mice, followed by PBS or drug administration from day 7 to day 21 (Figure S7A, Supporting Information). T26 treatment markedly suppressed lung metastasis, as evidenced by a significant reduction in metastatic nodules compared with control groups, likely due to a stronger systemic antitumor immune response (Figure S7B–D, Supporting Information). Moreover, T26 significantly prolonged the survival of colorectal cancer metastatic mice without affecting body weight (Figure S7E,F, Supporting Information). Collectively, these findings demonstrate that T26 effectively elicits systemic antitumor immunity, thereby preventing tumor metastasis and improving overall survival.

Notably, in the MC38 model, the prodrug T26 exhibited more pronounced tumor regression than the direct combination of MSA‐2 and JHU083 (Figure S7G–J, Supporting Information). This observation further supports that T26 enables the spatial and temporal synergy of JHU083 and MSA‐2, thereby exerting potent antitumor activity. Furthermore, to evaluate the essential role of STING signaling in mediating the antitumor activity of T26, we co‐administered the STING inhibitor H‐151 alongside T26 (Figure S7K, Supporting Information). The results showed that H‐151 markedly reversed the antitumor efficacy of T26, confirming that the in vivo antitumor activity of T26 is STING‐dependent (Figure S7L–N, Supporting Information).

PDOs serve as physiologically relevant ex vivo models that recapitulate the architectural and molecular features of the original tumors, thereby offering a valuable platform for assessing personalized therapeutic responses. To further evaluate the efficacy of T26 in human CRC, we treated CRC PDOs with T26. T26 administration significantly suppressed the growth of CRC PDOs (Figure 3K). Histological analysis by hematoxylin and eosin (H&E) staining, along with immunofluorescence staining for Ki67, revealed that T26 markedly reduced cell proliferation compared to JHU083 and MSA‐2 (Figure 3L).

Altogether, these findings reveal that oral administration of T26 significantly suppresses tumor growth and prolongs survival in both MC38 and CT26 colorectal cancer models.

### 2.4 T26 Suppresses Tumor Growth by Activating Dendritic Cells and CD8^+^ T Cells

**Figure 4:**
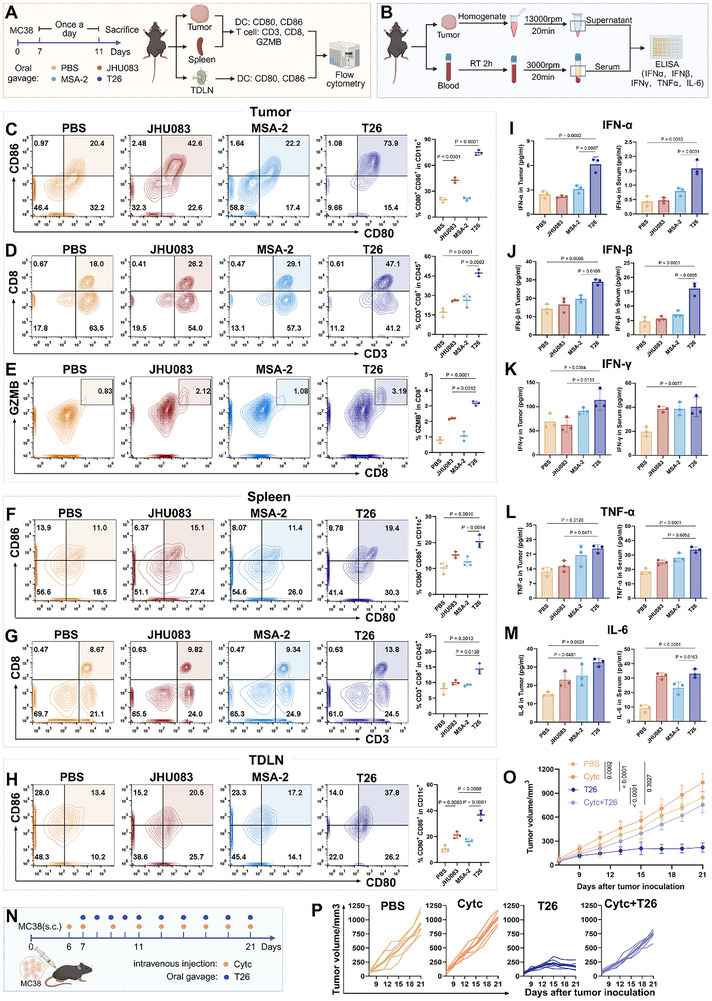
T26 enhances antitumor immunity through DC activation. (A) Experimental workflow for analyzing immune cell populations. C57BL/6 and BALB/c mice were subcutaneously inoculated with 1 × 10^6^ MC38 and CT26 cells, respectively, into the right flank on day 0. Once tumors reached approximately 80 mm^3^, mice were randomly assigned to receive either JHU083 or MSA‐2 at 1 mg kg^−1^ day^−1^ or T26 at 1.5 mg kg^−1^ day^−1^ from day 7 to 11. After treatment, tumors, spleens, and draining lymph nodes were harvested for flow cytometry analysis. (B) Experimental design for ELISA quantification of pro‐inflammatory cytokines in serum and tumors in MC38 or CT26 bearing mice following PBS, JHU083, MSA‐2, or T26 treatment. (C) Flow cytometry analysis of mature DCs (CD80⁺CD86⁺) in MC38 tumors (*n* = 3). (D) Flow cytometry analysis of CD8⁺ T cells (CD3⁺CD8⁺) in MC38 tumors (*n* = 3). (E) Flow cytometry analysis of GZMB expression in tumor‐infiltrating CD8⁺ T cells in MC38 tumors (*n* = 3). (F) Flow cytometry analysis of mature DCs (CD80⁺CD86⁺) in spleens (*n* = 3). (G) Flow cytometry analysis of CD8⁺ T cells (CD3⁺CD8⁺) in spleens (*n* = 3). (H) Flow cytometry analysis of mature DCs (CD80⁺CD86⁺) in TDLNs (*n* = 3). (I–M) Tumor and serum levels of IFN‐α, IFN‐β, IFN‐γ, TNF‐α, IL‐6 in MC38‐bearing mice (*n* = 3). (N) C57BL/6 mice with MC38 tumor were pretreated (i.v.) with either 5 mg per mouse of Cytc or PBS before other treatment and maintained the injection every other day for two weeks to deplete DCs. (O,P) Tumor growth of MC38 tumors in C57BL/6 mice with different treatment (*n* = 6). Error bars represent means ± SD. Differences between groups were tested using one‐way ANOVA followed by Tukey's multiple comparisons test, or unpaired Student's *t*‐test.

DCs are central orchestrators of antitumor immunity, primarily through capturing tumor‐associated antigens and presenting them to CD8⁺ cytotoxic T lymphocytes, thereby promoting effective immune responses.^[^ ^2^, ^3^, ^32^, ^33^ ^]^ To assess whether T26 suppresses tumor progression by enhancing DC maturation and CD8⁺ T cell activation, we conducted immunophenotypic analyses in MC38 and CT26 tumor‐bearing mice treated with T26 or control agents. Tumors, spleens, and tumor‐draining lymph nodes (TDLNs) were harvested post‐treatment and analyzed by flow cytometric analysis (**Figure** **4**A).

In the MC38 model, T26 treatment significantly increased the proportion of mature DCs in tumors by 54.67%, 31.83%, and 53.17% compared to PBS, JHU083, and MSA‐2 groups, respectively (Figure 4C). Consistent with enhanced antigen presentation, CD8⁺ T cells were markedly expanded in T26‐treated tumors, with increases of 30.2%, 21.17%, and 20.93% compared to the respective controls (Figure 4D). Importantly, these CD8⁺ T cells displayed elevated Granzyme B (GZMB) expression, a critical effector molecule for cytotoxic activity (Figure 4E). Beyond the tumor site, T26 treatment significantly promoted DC maturation in secondary lymphoid organs, including the spleen and TDLN, and increased the number of splenic CD8⁺ T cells, suggesting the establishment of systemic immune activation (Figure 4F–H).

To assess the functional implications of T26‐induced immune activation, we quantified cytokine production in tumor tissues and serum (Figure 4B). ELISA analysis revealed that T26 treatment markedly elevated levels of type I interferons (IFN‐α and IFN‐β) in both tumor tissues and serum. Additionally, key pro‐inflammatory cytokines, including IFN‐γ, TNF‐α, and IL‐6, were significantly elevated (Figure 4I–M). These results indicate robust activation of innate and adaptive immune signaling following T26 administration.

Cytochrome C (Cytc) is an agent that has been demonstrated to effectively deplete DCs.^[^ ^34^, ^35^ ^]^ To further determine whether DCs are functionally required for T26‐mediated tumor suppression, we selectively depleted DCs using Cytc. As expected, Cytc treatment significantly reduced DC populations in tumors, TDLNs, and spleens (Figure S8A–C, Supporting Information). In the absence of DCs, the therapeutic efficacy of T26 was nearly abolished (Figure 4N–P; Figure S8D, Supporting Information), underscoring the indispensable role of DCs in mediating the immune effects of T26. Importantly, no systemic toxicity was observed, as indicated by stable body weight in the T26 and Cytc co‐treated mice (Figure S8E, Supporting Information).

Parallel studies in the CT26 model yielded consistent results. T26 significantly increased the proportion of mature DCs in tumors, spleens, and TDLNs, and enhanced both the frequency and functional activity of intratumoral CD8⁺ T cells, including elevated GZMB expression. In addition, T26 increased the frequency of CD8⁺ T cells in the spleen (Figure S9A–F, Supporting Information). Additionally, T26 elevated IFN‐α and IFN‐β levels in both tumors and serum (Figure S9G,H, Supporting Information), along with increased secretion of TNF‐α, IFN‐γ, and IL‐6 (Figure S9I–K, Supporting Information).

To determine whether T26 exerts antitumor immunity through immune cells beyond DCs and CD8⁺ T cells, we established an MC38 tumor‐bearing mouse model and analyzed macrophages, NK cells, and CD4⁺ T cells by flow cytometry following five consecutive days of treatment (Figure S10A, Supporting Information). The results revealed that T26 increased the M1/M2 ratio of macrophages compared with the control group, although no significant difference was observed relative to JHU083, suggesting that macrophage modulation is not the primary reason underlying the superior efficacy of T26 over JHU083 or MSA‐2. In addition, T26 exerted no significant effects on CD4⁺ T cells or NK cells (Figure S10B–E, Supporting Information).

Together, these results demonstrate that T26 promotes robust DC maturation and CD8⁺ T cell cytotoxic activity across both tumor and peripheral lymphoid tissues, accompanied by elevated type I interferon signaling and pro‐inflammatory cytokine production, ultimately contributing to effective tumor suppression in vivo.

### 2.5 MC38 Tumor Cells Promote DCs Maturation through Vesicle‐Mediated Communication

**Figure 5:**
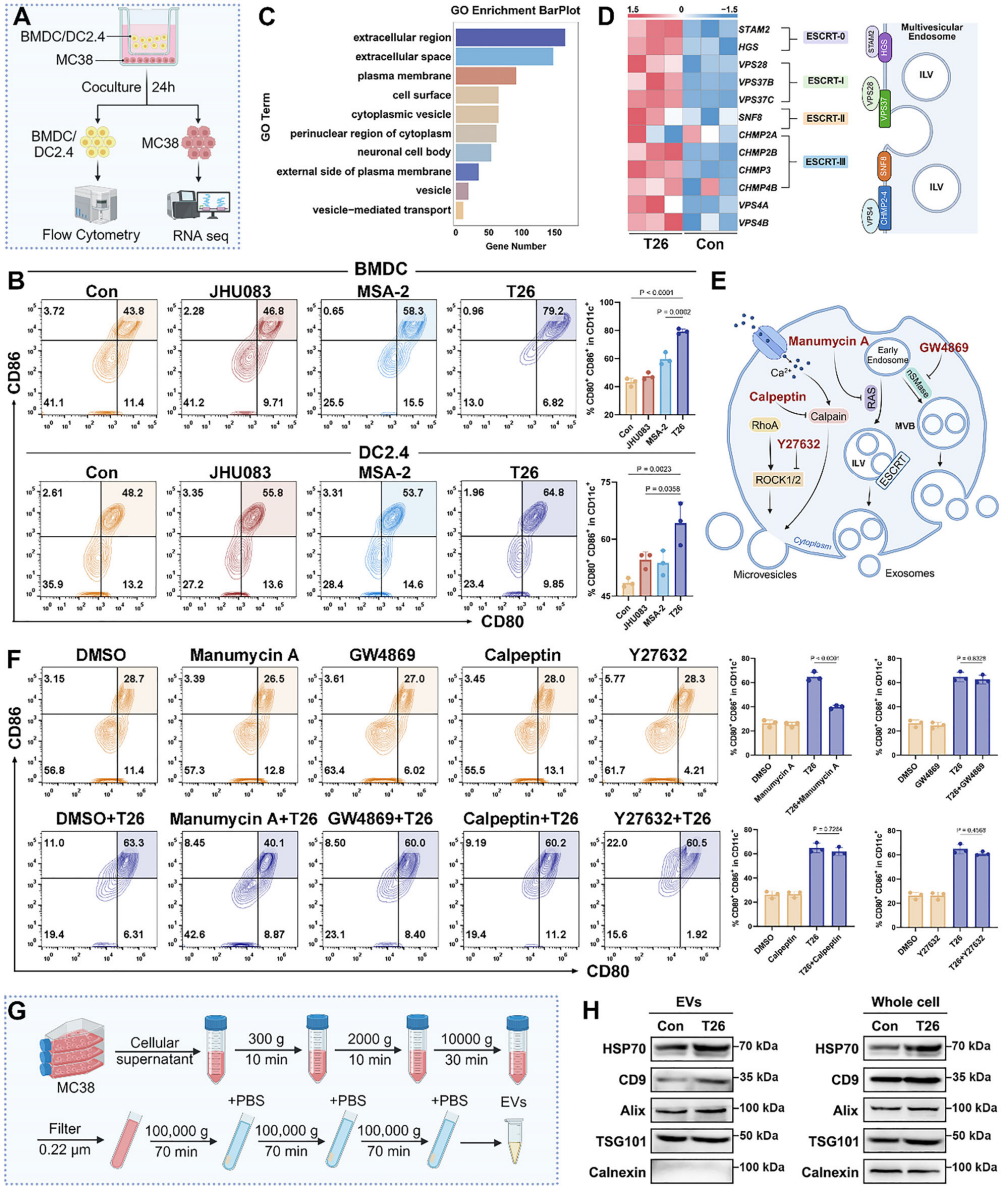
Tumor‐derived extracellular vesicles mediate DC activation upon T26 treatment. (A) Schematic of co‐culture experiments of MC38 and DCs. (B) Flow cytometry analysis of DC maturation in co‐culture with MC38 cells treated with DMSO, JHU083 (5 µm), MSA‐2 (5 µm), or T26 (5 µm) for 24 h (*n* = 3). (C) GO enrichment analysis of differentially expressed genes in response to T26‐treated MC38 cells. (D) Schematic illustrating the ESCRT machinery and a heatmap of gene expression associated with the ESCRT. (E) Schematic illustration of the action mechanisms of various vesicle inhibitors. (F) Maturation of BMDCs after treatment of MC38 and BMDCs co‐culture system with different vesicle inhibitors (*n* = 3). (G) Schematic illustration of EV isolation from MC38 culture supernatants. (H) Western blot analysis of EV markers and endoplasmic reticulum marker in isolated vesicles and whole cell lysates. Error bars represent means ± SD. Differences between groups were tested using one‐way ANOVA followed by Tukey's multiple comparisons test, or unpaired Student's *t*‐test.

Given that T26 treatment not only inhibits tumor cell proliferation but also enhances DCs maturation, we next investigated whether T26 could facilitate immunological crosstalk between tumor cells and DCs. To explore this, we co‐cultured MC38 tumor cells pretreated with different compounds (JHU083, MSA‐2, and T26) together with DCs and assessed their maturation status (**Figure** **5**A). Compared to the control group, the proportion of mature BMDCs was significantly increased in the T26‐treated co‐culture system. Furthermore, BMDCs in the T26‐treated group exhibited significantly higher levels of maturation compared to those treated with JHU083 or MSA‐2. Similar results were observed in co‐cultures involving the DC2.4 cell line (Figure 5B), suggesting that T26 enhances DC maturation at least in part through its effects on tumor cells. The significant increase in MHC‐I expression in the T26‐treated group demonstrates that T26 effectively enhances the antigen‐presenting capacity of dendritic cells (Figure S10F,G, Supporting Information).

To elucidate the molecular mechanisms underlying this tumor‐to‐DC communication, we performed RNA sequencing on MC38 cells following T26 treatment. GO enrichment analysis revealed significant upregulation of pathways related to EVs biogenesis and secretion, including annotations such as neuronal cell body, plasma membrane, cytoplasmic vesicle, the external side of the plasma membrane, and vesicle‐mediated transport (Figure 5C). These findings suggest that T26 may influence DC maturation via tumor cell‐derived EVs. Furthermore, we examined the gene expression profiles of key regulators involved in EVs formation, particularly components of the endosomal sorting complexes required for transport (ESCRT). T26 treatment led to marked upregulation of genes from multiple ESCRT subcomplexes, including ESCRT‐0 (e.g., *STAM2*, *HGS*), ESCRT‐I (e.g., *VPS28*, *VPS37B*, *VPS37C*), ESCRT‐II (e.g., *SNF8*), and ESCRT‐III (e.g., *CHMP2A*, *CHMP2B*, *CHMP3*, *CHMP4B*) (Figure 5D), further implicating enhanced EV production as a downstream effect of T26 treatment in tumor cells.^[^ ^36^, ^37^ ^]^

To directly test whether EVs mediate this effect, we employed a panel of pharmacological inhibitors targeting specific vesicle biogenesis pathways. EVs are primarily classified into microvesicles and exosomes.^[^ ^38^ ^]^ Calpeptin and Y27632 were used to block microvesicle release, while Manumycin A and GW4869 were applied to inhibit exosome production (Figure 5E). GW4869, which inhibits exosome release by targeting neutral sphingomyelinase (nSMase), showed no significant effect on T26‐induced maturation of BMDC. Similarly, Calpeptin and Y27632, which inhibit microvesicle formation and ROCK‐mediated cytoskeletal remodeling, respectively, had minimal impact on T26‐induced BMDC maturation. In contrast, only Manumycin A—which targets Ras signaling and impairs exosome formation—significantly reduced the T26‐induced maturation of BMDCs (Figure 5F).^[^ ^39^, ^40^, ^41^, ^42^ ^]^ These results suggest that T26 enhances DC activation primarily by promoting the Ras‐dependent formation of exosomes from tumor cells.

To further validate this mechanism, we isolated EVs from T26‐treated MC38 cells using ultracentrifugation (Figure 5G). Western blot analysis confirmed the enrichment of established exosome markers in the EV fractions, including TSG101, HSP70, CD9, and Alix. Meanwhile, the negative marker calnexin was detected only in whole‐cell lysates, not in EVs (Figure 5H), indicating the high purity of the isolated EVs and fulfilling the fundamental criteria outlined in the Minimal Information Guidelines for Extracellular Vesicle Studies (MISEV2018).^[^ ^43^ ^]^ Notably, T26 treatment markedly increased the abundance of EV markers, supporting the conclusion that T26 enhances EV production in tumor cells. These findings collectively demonstrate that T26 reprograms tumor cells to increase EV‐mediated immunostimulatory signaling, thereby promoting DC maturation and enhancing antitumor immunity.

In summary, these data demonstrate that T26 promotes dendritic cell maturation by reprogramming MC38 tumor cells to enhance exosome biogenesis, primarily via activation of Ras‐dependent exosome signaling pathways.

### 2.6 T26 Modulates Vesicles Secreted by Tumor Cells to Augment Immune Activation and Suppress Tumor Progression

Recent studies have demonstrated that engineered or stress‐induced tumor‐derived EVs can activate innate and adaptive immunity, and in some cases, elicit protective antitumor responses when used as cell‐free vaccines or therapeutic agents.^[^ ^44^, ^45^, ^46^, ^47^ ^]^ Building on these insights, and based on our above findings that T26 reprograms tumor cells to enhance EV biogenesis via Ras‐dependent signaling, we sought to determine whether EVs derived from T26‐treated tumor cells possess intrinsic immunostimulatory or antitumor activity independent of viable tumor cells.

**Figure 6:**
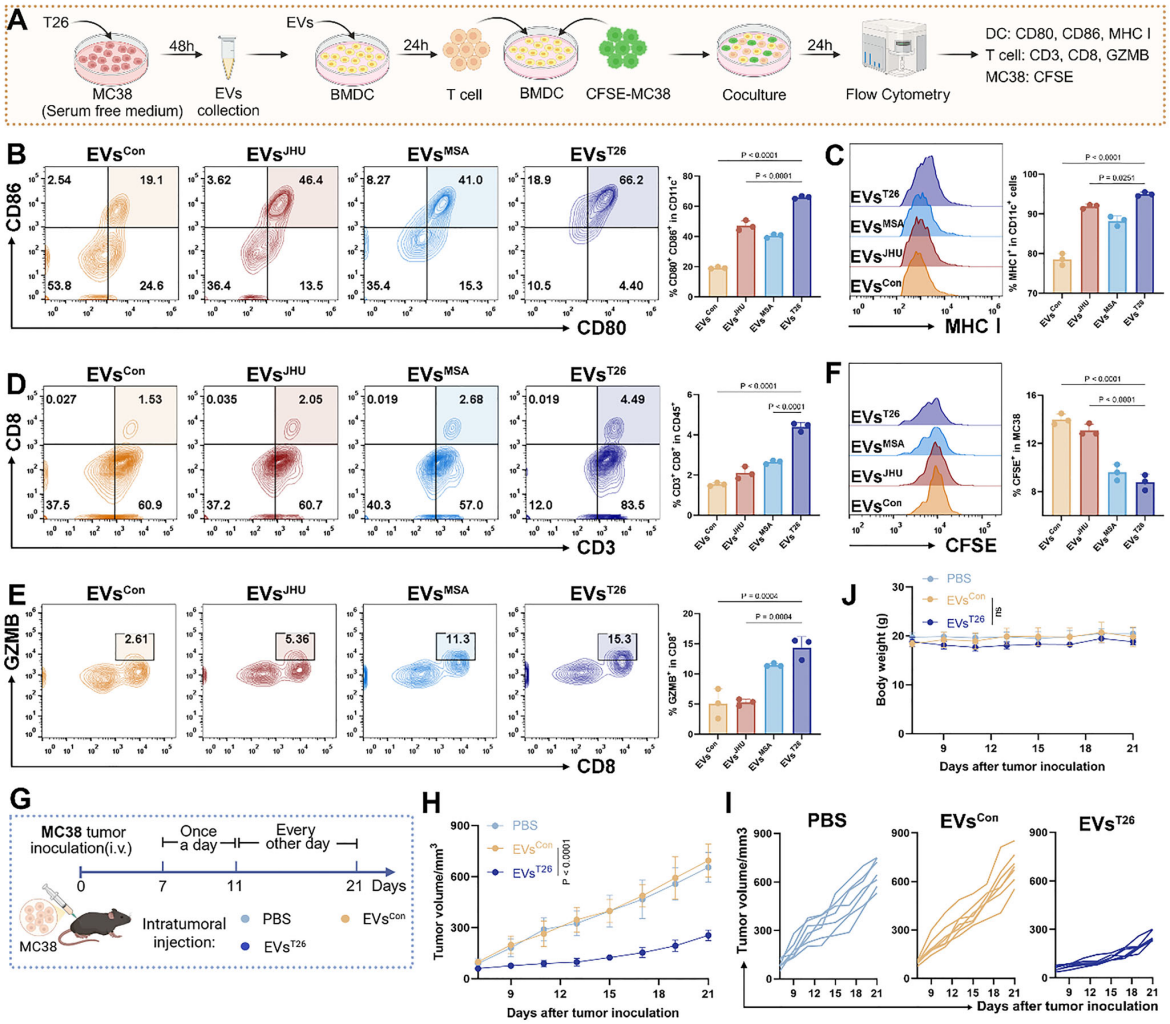
T26 reprograms tumor‐derived extracellular vesicles to enhance immune responses. (A) Experimental workflow for flow cytometry analysis: BMDCs were co‐cultured with CFSE‐labeled MC38 cells and T cells, and treated with EVs isolated from MC38 cells that had been treated with 5 µm MSA‐2, 5 µm JHU083, or 5 µm T26 for 48 h. (B) Flow cytometry analysis of DCs upon exposure to EVs^Con^, EVs^JHU^, EVs^MSA^, and EVs^T26^ (*n* = 3). (C) Flow cytometry analysis of MHC I antigen presentation in DCs incubated with EVs^Con^, EVs^JHU^, EVs^MSA^, and EVs^T26^ (*n* = 3). (D) Flow cytometry analysis of CD8⁺ T cell frequency expression after co‐culturing with BMDCs treated with EVs^Con^, EVs^JHU^, EVs^MSA^, and EVs^T26^ (*n* = 3). (E) Flow cytometry analysis of GZMB expression after co‐culturing with BMDCs treated with EVs^Con^, EVs^JHU^, EVs^MSA^, and EVs^T26^ (*n* = 3). (F) The killing of CFSE‐labeled MC38 following CD8⁺ T cell activation by EVs‐primed DCs (*n* = 3). (G) Schematic of the experimental regimen for intratumoral injection. MC38 tumor‐bearing mice received intratumoral injections of 100 µL PBS or EVs (20 µg in 100 µL PBS daily, days 7–11; 10 µg in 100 µL PBS every other day, days 12–21). (H,I) Tumor growth of MC38 tumors in C57BL/6 mice after intratumoral injection of PBS, EVs^Con^, and EVs^T26^ (*n* = 7). (J) The body weight changes of mice after intratumoral injection of PBS, EVs^Con^, and EVs^T26^ (*n* = 7). Error bars represent means ± SD. Differences between groups were tested using one‐way ANOVA followed by Tukey's multiple comparisons test, or unpaired Student's *t*‐test.

To investigate this, we isolated vesicles from MC38 cells treated with EVs control, JHU083, MSA‐2, or T26 (referred to as EVs^Con^, EVs^JHU^, EVs^MSA^, and EVs^T26^, respectively). BMDCs were incubated with each EV subset for 24 h, followed by co‐culture with CFSE‐labeled MC38 tumor cells and CD8⁺ T cells. Immune responses were evaluated by flow cytometry (**Figure** **6**A). The results revealed that EVs^T26^ robustly enhanced BMDC maturation, with a 46.83% increase compared to EVs^Con^ (Figure 6B). Additionally, MHC I antigen expression on BMDCs was markedly elevated in the EVs^T26^ group, indicating enhanced antigen presentation capacity (Figure 6C).

We next examined downstream effects on T cells. Co‐cultures exposed to EVs^T26^ exhibited an increased proportion of CD8⁺ T cells (up by 2.86% versus EVs^Con^; Figure 6D), along with significantly elevated expression of GZMB, a critical effector molecule for cytotoxic activity (Figure 6E). Notably, the number of CFSE‐labeled MC38 tumor cells was substantially reduced in the EVs^T26^ group (Figure 6F), indicating effective T cell‐mediated cytotoxicity. These results demonstrated that EVs from T26‐treated tumor cells not only enhance DC maturation and antigen presentation but also potentiate CD8⁺ T cell activation and tumor cell killing.

Based on these in vitro findings, we next evaluated the antitumor potential of EVs^T26^ in vivo. C57BL/6 mice bearing MC38 tumors received intratumoral injections of EVs^T26^ according to the schedule shown in Figure 6G. Strikingly, EVs^T26^ treatment significantly inhibited tumor growth, while having negligible effects on body weight (Figure 6H–J), indicating both efficacy and tolerability.

To elucidate the mechanism underlying the alteration of EV contents upon T26 treatment, we analyzed the transcriptomic profiles of MC38 cells exposed to T26. The results revealed a significant enrichment of pathways associated with immunogenic cell death (ICD), including the intrinsic apoptotic signaling pathway in response to endoplasmic reticulum stress, high mobility group box 1 binding pathway (HMGB1), ATP transmembrane transporter activity pathway, ATP biosynthetic process pathway, adaptive immune response pathway, and toll‐like receptor signaling pathway (Figure S11A, Supporting Information). Consistent with this, GSEA analysis revealed that several ICD‐associated pathways were markedly upregulated, suggesting that T26‐treated EVs may be enriched in damage‐associated molecular patterns (DAMPs) (Figure S11B–E, Supporting Information).

ICD is defined as a process in which tumor cells, upon external stimulation, switch from a non‐immunogenic to an immunogenic state.^[^ ^48^, ^49^ ^]^ During this process, tumor cells release a series of DAMPs, such as adenosine triphosphate (ATP), calreticulin (CRT), and HMGB1. Previous studies have reported that ICD in tumor cells can be transmitted via tumor‐derived EVs to immune cells, thereby establishing intercellular crosstalk.^[^ ^44^ ^]^ Based on these observations, we hypothesized that T26 promotes the release of EVs enriched in ICD‐related molecules. To further test this hypothesis, we isolated EVs secreted by MC38 cells under Control, JHU083, MSA‐2, and T26 treatments and examined their protein composition as well as DAMP content. Western blot analysis demonstrated that ICD markers HSP70 and CRT were significantly upregulated in EVs^T26^ compared with other groups (Figure S11F, Supporting Information). Moreover, ATP and HMGB1 were markedly enriched in EVs^T26^, as determined using an ATP assay kit and an HMGB1 ELISA kit, respectively (Figure S11G,H, Supporting Information). Together, these findings indicate that T26 facilitates the release of EVs carrying ICD‐related molecules, which may subsequently mediate downstream immune activation.

Altogether, these findings support the conclusion that T26 reprograms tumor cells to secrete immunostimulatory EVs that enhance DC maturation, promote CD8⁺ T cell activation, and facilitate effective tumor control both in vitro and in vivo.

### 2.7 Synergistic Inhibition of Tumor Growth by T26 in Combination with Chemotherapy and Immunotherapy

Immunotherapy and chemotherapy remain critical components in the treatment of CRC, but their effectiveness is often hindered by the immunosuppressive and metabolically hostile TME.^[^ ^50^, ^51^, ^52^ ^]^ Immune checkpoint inhibitors (ICIs), such as PD‐1/PD‐L1 blockade, have shown clinical benefits in select patients with microsatellite height instability (MSI‐H) or deficient mismatch repair (dMMR) tumors.^[^ ^52^, ^53^, ^54^, ^55^ ^]^ However, most CRCs, particularly microsatellite stable (MSS) or proficient mismatch repair (pMMR) subtypes, exhibit poor responsiveness to these therapies.^[^ ^56^ ^]^ The absence of CD8^+^ T cell infiltration within the TME is associated with a lack of response to PD‐1/PD‐L1 blockade therapy.^[^ ^57^, ^58^ ^]^ Against this backdrop, we sought to evaluate whether T26 could enhance tumor responsiveness to existing therapies and extend their efficacy in otherwise refractory settings.

**Figure 7:**
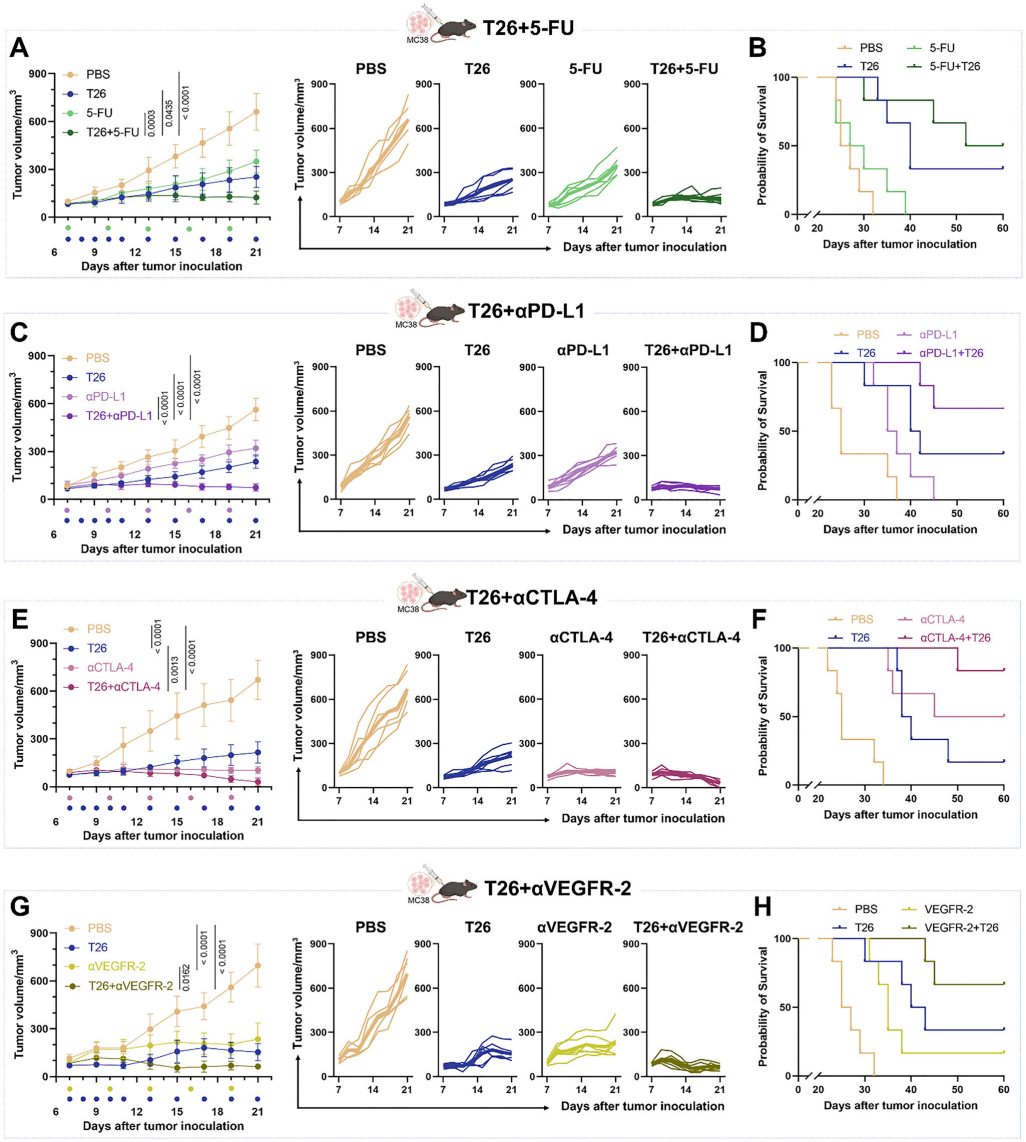
Synergistic tumor suppression by T26 in combination therapies. (A) Combined treatment with T26 (intragastric gavage, 1 mg kg^−1^ day^−1^ from day 7 to 11, followed by 0.3 mg kg^−1^ every other day from day 12 to 21) and 5‐FU (intraperitoneal injection, 50 mg kg^−1^, every three days from day 7 to 21) in MC38 tumor‐bearing mice. Synergistic efficacy and tumor growth curves of the 5‐FU+T26 group were compared with those of the PBS‐treated, T26‐treated, and 5‐FU‐treated groups (*n* = 6). (B) Kaplan–Meier survival analysis of MC38‐bearing mice with different treatment (*n* = 6). (C) Combined treatment with T26 (intragastric gavage, 1 mg kg^−1^ day^−1^ from day 7 to 11, followed by 0.3 mg kg^−1^ every other day from day 12 to 21) and αPD‐L1 (intraperitoneal injection, 2 mg kg^−1^, every three days from day 7 to 21) in MC38 tumor‐bearing mice. Synergistic efficacy and tumor growth curves of the αPD‐L1+T26 group were compared with those of the PBS‐treated, T26‐treated, and αPD‐L1‐treated groups (*n* = 6). (D) Kaplan–Meier survival analysis of MC38‐bearing mice with different treatment (*n* = 6). (E) Combined treatment with T26 (intragastric gavage, 1 mg kg^−1^ day^−1^ from day 7 to 11, followed by 0.3 mg kg^−1^ every other day from day 12 to 21) and αCTLA‐4 (intraperitoneal injection, 10 mg kg^−1^, every three days from day 7 to 21) in MC38 tumor‐bearing mice. Synergistic efficacy and tumor growth curves of the αCTLA‐4+T26 group were compared with those of the PBS‐treated, T26‐treated, and αCTLA‐4‐treated groups (*n* = 6). (F) Kaplan–Meier survival analysis of MC38‐bearing mice with different treatment (*n* = 6). (G) Combined treatment with T26 (intragastric gavage, 1 mg kg^−1^ day^−1^ from day 7 to 11, followed by 0.3 mg kg^−1^ every other day from day 12 to 21) and αVEGFR‐2 (intraperitoneal injection, 40 mg kg^−1^, every three days from day 7 to 21) in MC38 tumor‐bearing mice. Synergistic efficacy and tumor growth curves of the αVEGFR‐2+T26 group were compared with those of the PBS‐treated, T26‐treated, and αVEGFR‐2‐treated groups (*n* = 6). (H) Kaplan–Meier survival analysis of MC38‐bearing mice with different treatment (*n* = 6). Error bars represent means ± SD. Differences between groups were tested using one‐way ANOVA followed by Tukey's multiple comparisons test, or unpaired Student's *t*‐test.

We first examined the therapeutic interaction between T26 and 5‐FU. Although 5‐FU is a first‐line treatment for CRC, resistance frequently arises due to inadequate immune activation and adaptive tumor plasticity.^[^ ^59^, ^60^ ^]^ In MC38 tumor‐bearing mice, the combination treatment of T26 and 5‐FU significantly enhanced tumor growth inhibition compared to 5‐FU alone, without causing systemic toxicity or weight loss (**Figure** **7**A; Figure S12A, Supporting Information). Survival analyses further demonstrated that co‐treatment with T26 and 5‐FU significantly prolonged survival compared to 5‐FU monotherapy (Figure 7B). This additive effect likely results from T26's ability to alleviate immunometabolic constraints, facilitating chemotherapy‐induced immunogenic cell death and promoting downstream immune responses.

Next, we explored whether T26 could improve the efficacy of immune checkpoint blockade. The PD‐1/PD‐L1 axis plays a key role in immune evasion in CRC, and anti‐PD‐L1 (αPD‐L1) therapy has shown promise in certain CRC subsets, though responses are limited in MSS or pMMR tumors.^[^ ^55^, ^56^, ^61^ ^]^ In our model, co‐treatment with T26 and αPD‐L1 significantly enhanced tumor suppression compared to αPD‐L1 alone (Figure 7C; Figure S12B, Supporting Information), with no adverse effects on body weight. Furthermore, survival analysis showed that T26 combined with αPD‐L1 markedly prolonged survival compared to αPD‐L1 monotherapy (Figure 7D), suggesting that T26 promotes immune reactivation, likely by enhancing DC maturation and T cell activation within the TME.

We also investigated T26 in combination with anti‐CTLA‐4 (αCTLA‐4), another checkpoint inhibitor that facilitates T cell priming in early immune responses. While αCTLA‐4 therapy has shown activity in some patients, toxicity and resistance have limited its broader application in CRC.^[^ ^62^ ^]^ In MC38 tumors, T26 co‐treatment significantly improved the antitumor effect of αCTLA‐4 (Figure 7E; Figure S12C, Supporting Information) without inducing weight loss or overt toxicity. Survival analysis revealed a significant increase in survival when T26 was combined with αCTLA‐4 compared to αCTLA‐4 alone (Figure 7F), indicating that T26 helps overcome immunosuppressive barriers in the TME, improving the efficacy of CTLA‐4 blockade.

Furthermore, we assessed the synergy between T26 and anti‐VEGFR‐2 (αVEGFR‐2), an angiogenesis inhibitor that limits tumor vascularization. VEGFR‐2 is a critical therapeutic target in CRC due to its high expression on tumor cells. However, αVEGFR‐2 monotherapy shows limited efficacy, largely due to rapid resistance driven by compensatory signaling pathways.^[^ ^63^, ^64^ ^]^ We hypothesized that combining T26 with αVEGFR2 could concurrently inhibit multiple tumor‐promoting pathways, thereby reducing resistance and improving therapeutic outcomes. Indeed, the combination of T26 and αVEGFR‐2 significantly improved tumor control compared to αVEGFR‐2 monotherapy, with no adverse effects on body weight (Figure 7G; Figure S12D, Supporting Information). Moreover, co‐treatment significantly prolonged survival compared to αVEGFR‐2 alone (Figure 7H), suggesting that T26's ability to remodel the TME through metabolic and vascular modulation improves immune accessibility and enhances therapeutic outcomes.

Moreover, we evaluated the therapeutic efficacy of combining T26 with αPD‐L1 in the CT26 tumor model. Compared with αPD‐L1 monotherapy, the combination of T26 and αPD‐L1 resulted in markedly enhanced tumor suppression without causing adverse effects on body weight (Figure S12E,F, Supporting Information). These findings also indicate that T26 can potentiate the antitumor efficacy of αPD‐L1, thereby extending its therapeutic benefits to poorly immunogenic tumors.

Together, these results underscore the translational potential of T26 as an immunometabolic adjuvant capable of enhancing standard‐of‐care therapies across various therapeutic modalities. Whether combined with chemotherapeutic agents like 5‐FU, immune checkpoint inhibitors such as αPD‐L1 and αCTLA‐4, or anti‐angiogenic therapies like αVEGFR‐2, T26 consistently enhances therapeutic efficacy in vivo without compromising safety.

## 3 Discussion

**Figure 8:**
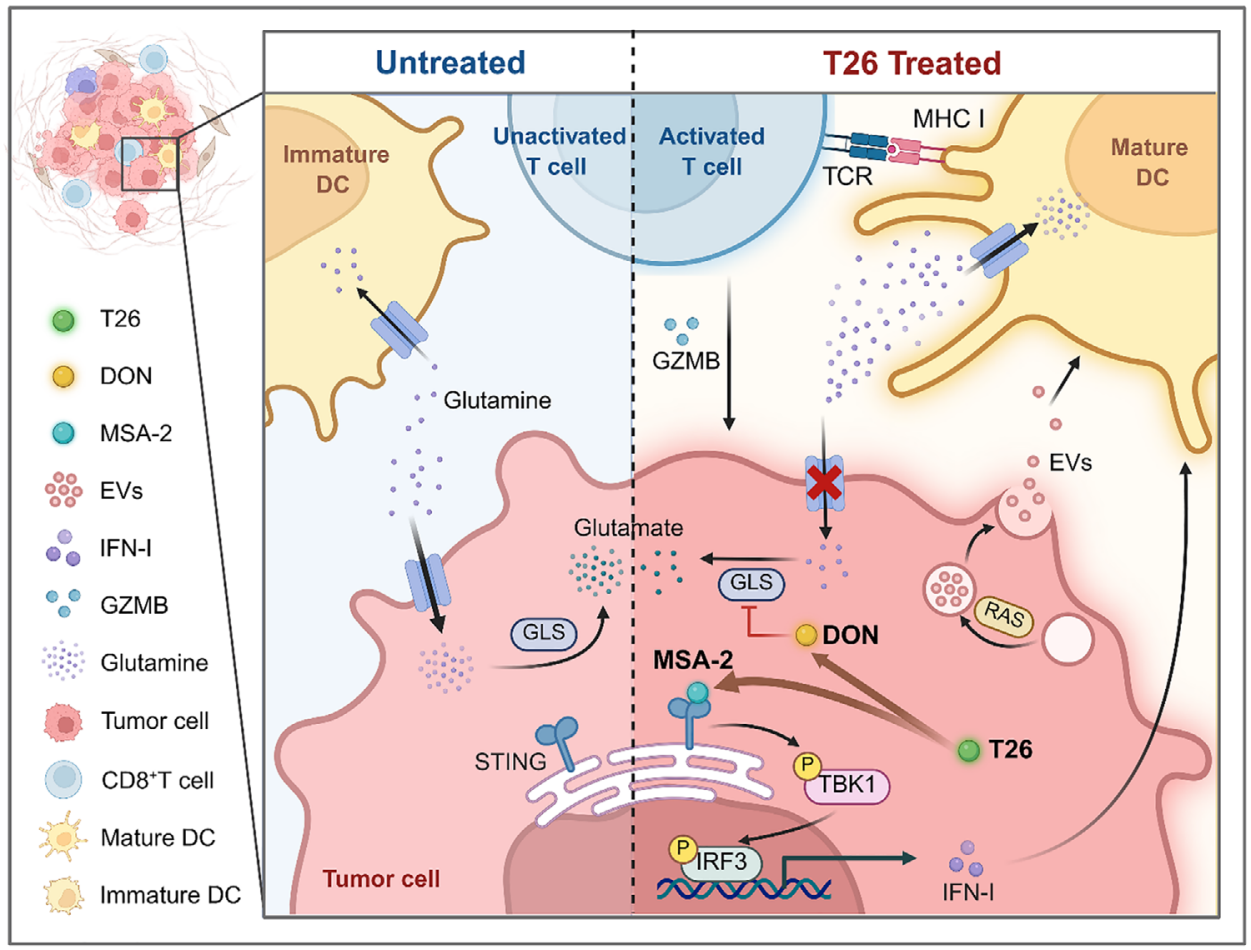
Schematic illustration of T26's antitumor mechanism.

In this study, we developed and characterized prodrug T26, a novel bifunctional immunomodulatory agent that integrates glutamine antagonism with STING pathway activation to remodel the TME and potentiate antitumor immunity. Our findings demonstrate that T26 simultaneously modulates tumor metabolism and innate immune signaling, thereby restoring DCs function, enhancing cytotoxic CD8⁺ T cell responses, and improving therapeutic outcomes in colorectal cancer models (**Figure** **8**).

Glutamine metabolism is central to supporting tumor cell proliferation while simultaneously restricting immune cell function by depleting nutrients critical for antigen presentation and effector activation.^[^ ^9^, ^65^ ^]^ Previous studies have shown that glutamine deprivation impairs DC maturation and T cell priming, thus facilitating immune escape.^[^ ^9^, ^12^ ^]^ While glutamine antagonists such as DON and its prodrug JHU083 partially alleviate these effects by limiting glutamine uptake in tumor cells, their capacity to fully restore immune function remains limited. Consistent with this, our data show that DON induces only partial DC maturation when co‐cultured with MC38 tumor cells, leaving a substantial fraction of DCs in an immature state. To elucidate the underlying mechanism, we performed transcriptomic profiling of BMDCs co‐cultured with tumor cells exposed to DON and identified suppression of STING‐related signaling as a key molecular constraint. Importantly, this mechanistic insight is mirrored in clinical colorectal cancer specimens, where tumors with elevated glutamine metabolic activity exhibit reduced STING expression and diminished mature DC infiltration. These findings underscore the clinical relevance of glutamine‐driven immunosuppression and highlight the need for coordinated metabolic and innate immune reprogramming.

To address this immunometabolic bottleneck, we rationally design prodrug T26 by conjugating JHU083 to the non‐nucleotide STING agonist MSA‐2 via a cleavable amide linker. This strategy enables synchronized, tumor‐selective release of both components, thereby maximizing intratumoral synergy while minimizing off‐target toxicity. Biochemical and structural analyses confirm successful synthesis, esterase‐responsive release kinetics, and functional retention of glutamine antagonism and STING activation. Cellular studies further demonstrate that T26 robustly induces activation of the STING pathway and inhibition of glutamine metabolism in tumor cells, validating its dual mechanistic design.

Therapeutically, T26 exhibits potent antitumor activity in vivo. Orally administered T26 significantly reduces tumor growth and prolongs survival in multiple murine colorectal cancer models, with no observable systemic toxicity or weight loss. Histological analysis reveals decreased tumor proliferation and increased p‐STING expression in tumor tissues, supporting efficient local activation of the STING pathway. These results align with recent efforts to develop orally available STING agonists and tumor‐selective metabolic modulators that are safe and systemically tolerable.

Effective DC activation is critical for initiating robust adaptive immune responses. Extending this paradigm, we demonstrate that T26 not only increases the frequency of mature DCs in the TME but also enhances their activation in spleens and TDLN. These changes are accompanied by increased infiltration, activation, and cytolytic capacity of CD8⁺ T cells, indicated by elevated GZMB expression and inflammatory cytokine production. Critically, depletion of DCs completely abrogates the antitumor efficacy of T26, directly confirming their indispensable role in mediating T26‐induced immune activation.

T26 reprograms tumor cells to enhance EVs production. Previous studies have shown that tumor‐derived EVs can either suppress or stimulate immunity depending on their molecular cargo and context. Our RNA sequencing data reveal that T26 treatment upregulates genes involved in the ESCRT machinery and vesicle trafficking, suggesting enhanced biogenesis of immunoregulatory EVs. Functionally, EVs derived from T26‐treated tumor cells significantly enhance DC maturation, antigen presentation, and subsequent CD8⁺ T cell activation. These EVs also mediate antitumor effects when administered intratumorally in vivo, establishing their relevance as active immune messengers. While the immunogenic potential of tumor‐derived EVs has been well‐documented, our findings provide direct evidence that pharmacologic reprogramming of EV content via T26 can redirect these vesicles toward immunostimulatory functions.

Importantly, we validate the therapeutic efficacy of T26 in CRC PDO, which closely recapitulates the structural and molecular heterogeneity of primary tumors. Treatment with T26 significantly suppresses PDO growth and reduces proliferation, as evidenced by Ki67 immunofluorescence and histological analysis. These findings underscore the translational relevance of T26 in clinically representative human models and support its potential applicability in precision oncology settings. Our data also indicate that prodrug T26 synergizes with multiple therapeutic modalities, including chemotherapy, checkpoint blockade, and anti‐angiogenic therapy. This is particularly relevant in CRC, where intrinsic and acquired resistance to monotherapies remains a major clinical challenge. Previous studies have shown that metabolic rewiring can sensitize tumors to chemotherapeutics, and STING activation may enhance the efficacy of 5‐FU, αPD‐L1, αCTLA‐4, and αVEGFR‐2 therapies. This combinatorial flexibility highlights the translational potential of T26 as an adjunct to diverse treatment strategies.

While tumors with MSI‐H or dMMR status are generally immunogenic and respond favorably to immune checkpoint blockade, the majority of CRCs are MSS or pMMR. Consequently, these tumors frequently resist current immunotherapies, largely due to their immunologically “cold” tumor microenvironment. Our findings demonstrate that T26 effectively mitigates metabolic immunosuppression, promotes dendritic cell maturation, and enhances the recruitment and activation of CD8⁺ T cells. These immunomodulatory actions directly counter the mechanisms of immune evasion commonly observed in MSS/pMMR tumors.

Together, our study establishes T26 as a mechanistically innovative and therapeutically versatile compound capable of reshaping the immunometabolic landscape of tumors. By simultaneously disrupting tumor cell metabolism, activating innate immune signaling, and reprogramming EV‐mediated intercellular communication, T26 offers a multipronged strategy to overcome key barriers limiting current cancer immunotherapy. Despite these promising results, further investigation into pharmacokinetics, long‐term safety, and the efficacy of T26 in other cancer types is required. Additionally, while we identify a functional role for T26‐induced EVs, the specific molecular cargo and downstream pathways responsible for immune activation remain to be elucidated.

## 4 Conclusion

In summary, our study establishes T26 as a mechanistically integrated and therapeutically promising immunometabolic agent that targets two fundamental vulnerabilities in the TME: glutamine‐driven metabolic immunosuppression and defective innate immune sensing. By simultaneously antagonizing glutamine metabolism and activating the STING pathway, prodrug T26 enhances dendritic cell maturation, promotes CD8⁺ T cell activation, and reprograms tumor‐derived extracellular vesicles to further propagate immune responses. These effects collectively reshape the immunological landscape of tumors and lead to robust antitumor efficacy in preclinical models. Importantly, T26 also exerted potent antitumor effects in CRC PDO, underscoring its clinical translatability in patient‐relevant preclinical models. Furthermore, the ability of T26 to synergize with chemotherapies, immunotherapies, and targeted therapy underscores its translational potential and provides a compelling rationale for its future clinical investigation in combination immunotherapy strategies. This work provides a conceptual and mechanistic foundation for the design of next‐generation immunometabolic therapies that integrate nutrient modulation with innate immune activation to overcome the metabolic barriers of the TME.

## 5 Experimental Section

### Study design

This study was designed to investigate the immunometabolic mechanisms by which glutamine antagonism and innate immune activation coordinate to modulate DC function and reshape the TME in CRC. A transwell co‐culture system was first employed in which MC38 tumor cells were treated with the glutamine antagonist DON to restrict glutamine uptake, thereby indirectly modulating nutrient availability for BMDCs. This setup revealed that glutamine supplement alone induced only partial DC maturation. To explore the underlying mechanism, transcriptomic profiling of BMDCs co‐cultured with DON‐treated tumor cells was performed, and suppression of STING pathway signaling was identified as a key limiting factor. Based on these findings, T26, a bifunctional prodrug in which JHU083 and STING agonist MSA‐2 are covalently linked via a cleavable amide bond, is designed and synthesized, enabling synchronized intratumoral release and dual targeting of glutamine metabolism and innate immune activation. RNA‐seq was further applied to T26‐treated MC38 tumor cells to identify immunomodulatory gene expression changes associated with T26 activity.

The in vivo antitumor efficacy of T26 was next evaluated in syngeneic MC38 and CT26 tumor models. Mice were treated with T26, JHU083, MSA‐2, or PBS, tumor progression, systemic toxicity, and immune infiltration were assessed. Flow cytometry and ELISA were used to characterize DC maturation and CD8⁺ T cell activation in both tumors and secondary lymphoid tissues. To determine the requirement for DCs in mediating T26's antitumor effects, cytochrome c‐induced depletion was used, which abolished therapeutic efficacy. It was then investigated whether tumor‐derived EVs contribute to DC activation. Pharmacological inhibition of EV biogenesis was first used to assess the role of extracellular vesicles in T26‐induced DC activation. Subsequently, EVs were isolated from T26‐treated MC38 cells and functionally validated through EV transfer assays and in vivo administration. These studies revealed that T26 reprograms tumor cells to produce immunostimulatory EVs that enhance DC maturation and CD8⁺ T cell responses. Finally, the combinatorial potential of T26 with checkpoint inhibitors, chemotherapy, and anti‐angiogenic agents was assessed to evaluate its translational applicability in therapeutic regimens.

### Cell Lines

All cell lines were maintained at 37 °C in a humidified incubator (ThermoFisher‐Forma 371) with 5% CO_2_. MC38 (The mouse colon adenocarcinoma cell line, Procell, CL‐0972, RRID: CVCL_B288) was purchased on January 9, 2024, and was derived from Female C57BL/6 mice. CT26 (The mouse colon carcinoma cell line, Procell, CL‐0071, RRID: CVCL_7256) was purchased on January 15, 2024, and was derived from BALB/c mice. DC2.4 (The mouse dendritic cell line, Procell, CL‐0545, RRID: CVCL_J409) was purchased on March 6, 2024, and was derived from C57BL/6 mice. MC38 cells were cultured in Dulbecco's Modified Eagle Medium (DMEM, high glucose, Sangon Biotech, Shanghai, E600003) supplemented with 10% fetal bovine serum (FBS) and 1% penicillin–streptomycin (100×, PS, Macgene, Beijing, CC004). CT26 and DC2.4 cells were maintained in RPMI‐1640 medium supplemented with 10% FBS and 1% PS. In this study, the cell lines involved were confirmed to be correct and free of contamination after short tandem repeat (STR) analysis and quality inspection.

### BMDC Isolation

Female C57BL/6 mice (6–8 weeks old) were euthanized by cervical dislocation and immersed in 75% ethanol to ensure sterility. The femur and tibia were dissected, and attached muscle and connective tissue were carefully removed using sterile scissors and forceps while preserving bone integrity. The bone marrow was flushed out using a sterile syringe filled with RPMI‐1640 medium and collected into a sterile centrifuge tube. The suspension was filtered through a 70‐µm cell strainer (Biofil, Guangzhou, CSS013070) and centrifuged at 2000 rpm for 5 min, after which the supernatant was discarded. The resulting cell pellet, containing both bone marrow cells and erythrocytes, was resuspended in erythrocyte lysis buffer (Tiangen, Beijing, RT122‐02) and incubated at room temperature for 5 min. The lysis reaction was quenched by adding 2–3 volumes of RPMI‐1640 medium, followed by centrifugation at 3000 rpm for 5 min. The pellet was resuspended in RPMI‐1640 complete medium supplemented with 20 ng mL^−1^ granulocyte‐macrophage colony‐stimulating factor (GM‐CSF, Sino Biological, Beijing, 51048‐MNAE) and 10 ng mL^−1^ interleukin‐4 (IL‐4, Sino Biological, Beijing, 51084‐MNAE). Cells were seeded at a density of 1 × 10⁶ cells mL^−1^ in six‐well plates and incubated at 37 °C with 5% CO_2_. The culture medium was replaced every three days, and BMDCs were fully differentiated by day 7–8.

### Animals

Female C57BL/6 and BALB/c mice (6–8 weeks old, 18–20 g) were purchased from Keaoke Biotechnology Co., Ltd. All animals were housed in a sterile environment with a controlled humidity of 40%–70% and a stable temperature of 19–23 °C under a 12‐h light/dark cycle. All experimental procedures were conducted in accordance with the ARRIVE guidelines (EU Directive 2010/63/EU) and approved by the Institutional Animal Care and Use Committee of Northwestern Polytechnical University.

### Flow Cytometry

For cell samples, an appropriate volume of antibody was incubated with cells at 4 °C for 40 min. For tumor tissue samples, tumors were digested in RPMI‐1640 medium containing 2 mg mL^−1^ collagenase I, 2 mg mL^−1^ collagenase IV, and 0.1 mg mL^−1^ DNase I at 37 °C for 30 min. The digested suspension was filtered through a 70‐µm cell strainer and centrifuged at 3000 rpm. The resulting cell pellet was resuspended and incubated with the appropriate antibodies.

For spleen samples, the spleens were mechanically dissociated and rinsed with sterile PBS until all tissue passed through the cell strainer. The cell suspension was centrifuged, and the resulting cells were treated with erythrocyte lysis buffer for 5 min. Then the cells can be incubated with the antibody. For draining lymph node samples, lymph nodes were mechanically dissociated and rinsed with sterile PBS until all tissue passed through the cell strainer. After centrifugation, the resulting cell pellet was prepared for antibody incubation. When intracellular staining was required, cells were first fixed for 30 min at room temperature, followed by staining with antibodies prepared in a permeabilization buffer and incubated for 40 min at 4 °C. A complete list of antibodies used is provided in Table S1 (Supporting Information).

### Cell Viability Assay

Five thousand cells per well were seeded into a 96‐well plate and incubated for 24 h. Subsequently, the cells were treated with varying concentrations of the test drug and further incubated for 48 h. Following drug exposure, 0.5 mg mL^−1^ solution of Methyl Thiazolyl Tetrazolium (MTT) (Adamas, Shanghai, 01269628) in incomplete medium was added to each well, and incubation continued for an additional 5 h. The medium was then carefully aspirated, and 200 µL of dimethyl sulfoxide (DMSO) (Beyotime, Shanghai, ST1276) was added to dissolve the formazan crystals. The plate was shaken at room temperature for 15 min while being protected from light. Absorbance at 490 nm was measured using a microporous plate multifunctional detector (Tecan−Tecan Spark, Switzerland).

### RNA‐seq

Total RNA was extracted from cells using Trizol reagent (Thermo Fisher, 15596018).RNA sequencing was performed by Lianchuan Biologicals using the Illumina X10 sequencing platform (Hangzhou, Zhejiang, China). Differentially expressed genes were identified based on the criteria of an absolute log2 fold change (|log2FC|) ≥ 1 and a q‐value < 0.05 (adjusted *p*‐value). Gene Ontology (GO) analysis and Gene Set Enrichment Analysis (GSEA) were conducted to characterize differentially expressed gene sets.

### Glutamine Content Assay

After removing the culture medium, cells were resuspended in 0.9% saline and homogenized. The homogenate was centrifuged at 10 000 g for 15 min at 4 °C, and the supernatant was collected for glutamine quantification using a Glutamine Assay Kit (Elabscience, Wuhan, E‐BC‐K853‐M).

### Immunofluorescence Staining

For cellular immunofluorescence staining, cells were fixed with 4% paraformaldehyde (PFA) (Servicebio, G1101) for 10 min, followed by three washes with PBS for 5 min each. Permeabilization was performed using PBS containing 0.2% Triton X‐100 for 10 min, followed by three additional PBS washes. To block nonspecific binding, cells were incubated with PBS containing 22.52 mg mL^−1^ glycine and 1% bovine serum albumin (BSA) for 30 min. Primary antibodies were applied and incubated overnight at 4 °C. After washing three times with PBS, secondary antibodies were incubated for 1 h at room temperature, followed by additional PBS washes. For tissue samples, paraffin‐embedded or frozen sections were stained using the same protocol. Images were acquired using a Confocal Laser Scanning Microscope (CLSM, OLYMPUS FV3000). Antibody details are listed in Table S1 (Supporting Information).

### Western Blot

Cell samples were resuspended in PBS, centrifuged at 3000 rpm for 5 min at 4 °C, and lysed in RIPA buffer supplemented with PMSF and a phosphatase inhibitor. The lysates were centrifuged at 12 000 rpm for 10 min at 4 °C, and the supernatant was collected for protein quantification using a BCA kit (Sangon Biotech, Shanghai, C503021). Protein samples were denatured by boiling in 5X loading buffer for 10 min at 95 °C, separated via sodium dodecyl sulfate–polyacrylamide gel electrophoresis (SDS‐PAGE), and transferred to a PVDF membrane using a constant current of 250 mA. Membranes were blocked with 5% non‐fat milk in TBST for 1 h at room temperature, followed by overnight incubation with primary antibodies at 4 °C. After three washes with TBST, membranes were incubated with secondary antibodies for 1 h at room temperature and washed again. Protein bands were detected using enhanced chemiluminescence (ECL) and visualized with a chemiluminescence imager. All antibodies are detailed in Table S1 (Supporting Information).

### Tumor Models

C57BL/6 and BALB/c mice were subcutaneously inoculated with 1 × 10^6^ MC38 and CT26 cells, respectively, into the right flank on day 0. Once tumors reached ≈80 mm^3^, mice were randomly assigned to receive either JHU083 or MSA‐2 at 1 mg kg^−1^ day^−1^ from day 7 to 11, followed by 0.3 mg kg^−1^ every other day from day 12 to 21, or T26 at 1.5 mg kg^−1^ day^−1^ from day 7 to 11, followed by 0.45 mg kg^−1^ every other day from day 12 to 21. The drugs were administered daily for the first 5 days and then every other day until day 21. During this period, body weight and tumor size were recorded every other day, and tumor volume was calculated using the formula (length × width^2^ / 2). On day 21, mice were euthanized, and tumors, hearts, livers, spleens, lungs, and kidneys were harvested for H&E staining and immunofluorescence analysis.

### Organoid Culture

Colorectal cancer patient‐derived organoids were purchased from D1Med (Shanghai, China). Organoids were resuspended in Matrigel (D23016‐0010, D1Med) that had been diluted with organoid culture medium (K211M01, D1Med) at a ratio of 3:7 (v/v). A total of 50 µL of the Matrigel mixture was seeded into each well of a 24‐well plate for 3D culture. After the Matrigel solidified, 500 µL of organoid culture medium was gently added to each well. Once the PDOs reached a diameter of ≈80 µm, drug treatments were initiated.

### Organoid staining

The organoid culture medium was carefully aspirated. Each well was rinsed with 1 mL of cold DPBS, and the Matrigel was dislodged by gentle pipetting. Organoids were transferred to 15 mL centrifuge tubes. After gentle pipetting to separate organoids from the surrounding Matrigel, the suspension was adjusted to volume with DPBS and centrifuged at 2500 rpm for 5 min at 4 °C. The supernatant was discarded, and the organoids were resuspended in DPBS, pipetted gently several times to remove residual Matrigel, and centrifuged again under the same conditions. The resulting organoids were fixed in 4% PFA at a volume exceeding tenfold of the pellet, gently mixed, and incubated overnight at 4 °C. For H&E staining, fixed organoids were pre‐embedded in 2% sodium alginate to preserve their 3D architecture, followed by dehydration, clearing, and paraffin embedding. Paraffin blocks were sectioned at a thickness of 4 µm, deparaffinized, rehydrated, and stained using standard H&E protocols. For immunofluorescence staining, fixed organoids were permeabilized and blocked, then incubated overnight at 4 °C with primary antibodies. The following day, the samples were incubated with fluorescently labeled secondary antibodies for 1 h at room temperature in the dark. Stained organoids were subsequently mounted onto glass slides and coverslipped for imaging.

### ELISA

Blood samples were collected from the orbital venous plexus of mice and allowed to clot at room temperature for 2 h. Serum was obtained by centrifugation at 3000 rpm for 20 min. ELISA assays were performed following the instructions of the manufacturers. The following ELISA kits were used: IFN‐α (ThermoFisher, BMS6027), IFN‐β (R&D Systems, MIFNB0), IL‐6 (Invitrogen, 88‐7064‐88), TNF‐α (Invitrogen, 88‐7324‐88), IFN‐γ (Invitrogen, 88‐7314‐88), HMGB1 (ThermoFisher, EEL102).

### Extracellular Vesicle Isolation

Extracellular vesicles were isolated via ultracentrifugation. MC38 cells were cultured in serum‐free DMEM until reaching 80–90% confluency. The conditioned medium was sequentially centrifuged at 300 g for 5 min, 2000 g for 10 min, and 10 000 g for 30 min to remove debris. The supernatant was then filtered through a 0.22 µm filter (Biofil, Guangzhou, FPE204030) before ultracentrifugation at 100 000 g for 70 min. The pellet was resuspended in PBS, followed by a second ultracentrifugation at 100 000 g for 70 min. The final particles were extracellular vesicles that were resuspended in PBS and stored at − 80 ° C for further analysis. Calpeptin (TargetMol, T6432) and Y27632 (TargetMol, T1870), Manumycin A (TargetMol, T16011), GW4869 (TargetMol, T3640).

### Characterization of Extracellular Vesicles

Extracellular vesicles were lysed in RIPA buffer supplemented with PMSF and a phosphatase inhibitor, followed by ice incubation for 30 min. After centrifugation at 12 000 rpm for 10 min at 4 °C, the supernatant was collected for protein quantification using a BCA assay. Western Blot could be performed after the protein was denatured by adding loading buffer and boiling.

### siRNA Transfection

Small interfering RNA (siRNA) targeting mouse STING was purchased from Tsingke Biotechnology Co. Three distinct siRNA duplexes were evaluated. The sequences of siRNAs are listed in Table S2 (Supporting Information). Stable MC38 cell lines were established by puromycin (Beyotime, Shanghai, ST551) selection following siRNA transfection.

### Cell Invasion Assay

Cell invasion was assessed using a Matrigel‐coated Transwell system. Briefly, the upper chambers were pre‐coated with diluted Matrigel (1 mg mL^−1^) and hydrated at 37 °C. MC38 cells were then resuspended in serum‐free medium and seeded into the upper chambers at a density of 10⁵ cells per well, while the lower chambers were filled with complete medium. The cells were treated with DMSO, JHU083, MSA‐2, or T26 for 48 h. Following incubation, non‐invaded cells on the upper surface were removed by swabbing, while the invaded cells on the lower membrane were fixed with paraformaldehyde, stained with crystal violet, and imaged using an inverted microscope.

### Scratch Wound Healing Assay

A scratch wound healing assay was performed to evaluate the migratory ability of MC38 cells. The cells were seeded in six‐well plates and cultured until a confluent monolayer was formed. A straight scratch was created in the monolayer using a p200 pipette tip, and the initial wound width was recorded under a microscope. The cells were then treated with DMSO, JHU083, MSA‐2, or T26 for 24 h. Following treatment, the same wound areas were re‐imaged to assess cell migration.

### Statistical Analysis

Data are presented as mean ± SD. The sample size (*n*) for each experiment is indicated in the corresponding figure legends. No data points were excluded, and no data transformations were applied unless otherwise stated. All data have been generated from at least three independent biological experiments, unless otherwise specified in the figure legends. Comparisons between two groups were performed using a two‐tailed unpaired Student's *t*‐test. For experiments involving more than two groups, one‐way analysis of variance (ANOVA) followed by Tukey's multiple comparisons test was applied. Statistical significance was defined as *p* < 0.05. All analyses were performed using GraphPad Prism version 9.0 (GraphPad Software, San Diego, CA, USA). The TOC graphic and supporting schematic illustrations were created using BioRender.com under an academic license.

### Ethics Approval

Ethics approval and consent to participate Human CRC specimens were obtained from Fudan University Shanghai Cancer Center. Experiments with human tumor tissue were conducted in accordance with approved institutional review board protocols (2407‐ZZK‐123). All patients provided written informed consent in accordance with institutional review board protocols. All animal experiments were granted by the Ethics Committee of Animal Experiment Center of Northwestern Polytechnical University Animal Care and Use Guidelines (202401131). Raw data for the bulk RNA‐seq analysis comparing mature and immature BMDCs have been deposited in the Gene Expression Omnibus (GEO) database under accession number GSE296658. Raw data for the bulk RNA‐seq analysis comparing T26‐treated and control MC38 cells have been deposited in the GEO database under accession number GSE297452.

## Conflict of Interest

The authors declare no conflict of interest.

## Author Contributions

B.Z., R.F., Y.H., and Y.C. contributed equally to this work. L.Y., L.L., M.Z., and G.W. conceived and designed the study. B.Z., R.F., Y.H., Ye C., X.L., W.W., J.D., J.Y., C.S., and Y.C. performed the experiments and developed the methodology. B.Z., R.F., and G.W. wrote the original draft. All authors discussed the results, contributed to manuscript revision, and approved the final version of the manuscript.

## Supporting information



Supporting Information

## Data Availability

The data that support the findings of this study are available from the corresponding author upon reasonable request.
