# Supplementary material for: Immunometabolic Rewiring of Dendritic Cells to Overcome Glutamine‐Driven Immune Suppression in Colorectal Cancer
Source: Adv Sci (Weinh). 2025 Oct 31;13(5):e13986. doi: 10.1002/advs.202513986 (PMC12849977; doi:10.1002/advs.202513986)
Supplement: Supplementary file 1 — Supporting Information [file ADVS-13-e13986-s001.docx]

**Supporting Information**

**Immunometabolic Rewiring of Dendritic Cells to Overcome Glutamine-Driven Immune Suppression in Colorectal Cancer**

*Bingjie Zhang, Renming Fan, Yongrui Hai, Ye Chen, Xintong Lu, Wenhui Wang, Jiarui Dou, Jiaxin Yan, Chang Su, Yue Chen, Le Yang*, Minggao Zhao*, Lei Liang*, and Gaofei Wei**

Bingjie Zhang, Renming Fan, Yongrui Hai and Ye Chen contributed equally to this work.

**Table S1. Antibodies used for IF, Western blot and flow cytometry**

| **Antibody** | **Manufacturer** | **Catalog** |
| --- | --- | --- |
| Anti-Vinculin | Abcam | ab129002, RRID:AB_11144129 |
| STING (D1V5L) Rabbit mAb | Cell Signaling Technology | 50494, RRID:AB_2799375 |
| Phospho-STING (Ser365) (D8F4W) Rabbit mAb | Cell Signaling Technology | 72971, RRID:AB_2799831 |
| Phospho-STING (Ser365) (D1C4T) Rabbit mAb | Cell Signaling Technology | 62912, RRID:AB_2799635 |
| TBK1/NAK (D1B4) Rabbit mAb | Cell Signaling Technology | 3504, RRID:AB_2255663 |
| Phospho-TBK1/NAK (Ser172) (D52C2) XP Rabbit mAb | Cell Signaling Technology | 5483, RRID:AB_10693472 |
| IRF-3 (D83B9) Rabbit mAb | Cell Signaling Technology | 4302, RRID:AB_1904036 |
| Phospho-IRF-3 (Ser396) (D6O1M) Rabbit mAb | Cell Signaling Technology | 29047, RRID:AB_2773013 |
| Goat Anti-Rabbit IgG H&L | Abcam | ab205718, RRID:AB_2819160 |
| Goat Anti-Rabbit IgG H&L(AlexaFluor®488) | Abcam | ab150077 , RRID:AB_2630356 |
| Alix | Proteintech | 12422-1-AP, RRID:AB_2162467 |
| CD9 | Proteintech | 20597-1-AP, RRID:AB_2878706 |
| HSP70 | Proteintech | 10995-1-AP, RRID:AB_2264230 |
| Calnexin | Proteintech | 10427-2-AP, RRID:AB_2069033 |
| **Antibody** | **Manufacturer** | **Catalog** |
| TSG101 | Proteintech | 28283-1-APRRID:AB_2881104 |
| PE anti-mouse CD80 Antibody | Biolegend | 104708, RRID:AB_313129 |
| FITC anti-mouse CD11c Antibody | Biolegend | 117306, RRID:AB_313775 |
| Alexa Fluor® 700 anti-human/mouse Granzyme B Recombinant Antibody | Biolegend | 372222, RRID:AB_2728389 |
| PE/Dazzle™ 594 anti-mouse CD45 Antibody | Biolegend | 103146, RRID:AB_2564003 |
| APC/Cyanine7 anti-mouse CD103 Antibody | Biolegend | 121432, RRID:AB_2566552 |
| PE anti-mouse H-2Kb/H-2Db Antibody | Biolegend | 114607, RRID:AB_313598 |
| CD86/B7-2 (GL-1) Rat mAb (APC Conjugate) | Cell Signaling Technology | 84393, RRID:AB_3665017 |
| CD3 (17A2) Rat mAb (APC Conjugate) | Cell Signaling Technology | 24265, RRID:AB_2798875 |
| CD8α (2.43) Rat mAb (PE Conjugate) | Cell Signaling Technology | 56984, RRID:AB_2799523 |
| CD4 (RM4-5) Rat mAb (FITC Conjugate) | Cell Signaling Technology | 96127, RRID:AB_2943473 |
| APC/Cyanine7 anti-mouse CD69 Antibody | Biolegend | 104526, RRID:AB_10679041 |
| Brilliant Violet 650™ anti-mouse NK-1.1 Antibody | Biolegend | 108736, RRID:AB_2563159 |
| FITC anti-mouse F4/80 Antibody | Biolegend | 123107, RRID:AB_893500 |
| PE anti-mouse CD206 (MMR) Antibody | Biolegend | 141706, RRID:AB_10895754 |

**Table S2. shRNA sequences targeting STING**

| **Target** | **Sequence** |
| --- | --- |
| Mouse shSTING#1 | CCGGATGATTCTACTATCGTCTTATCTCGAGATAAGACGATAGTAGAATCATTTTTTT |
| Mouse shSTING#2 | CCGGCAACATTCGATTCCGAGATATCTCGAGATATCTCGGAATCGAATGTTGTTTTTT |
| Mouse shSTING#3 | CCGGAGAGGTCACCGCTCCAAATATCTCGAGATATTTGGAGCGGTGACCTCTTTTTTT |


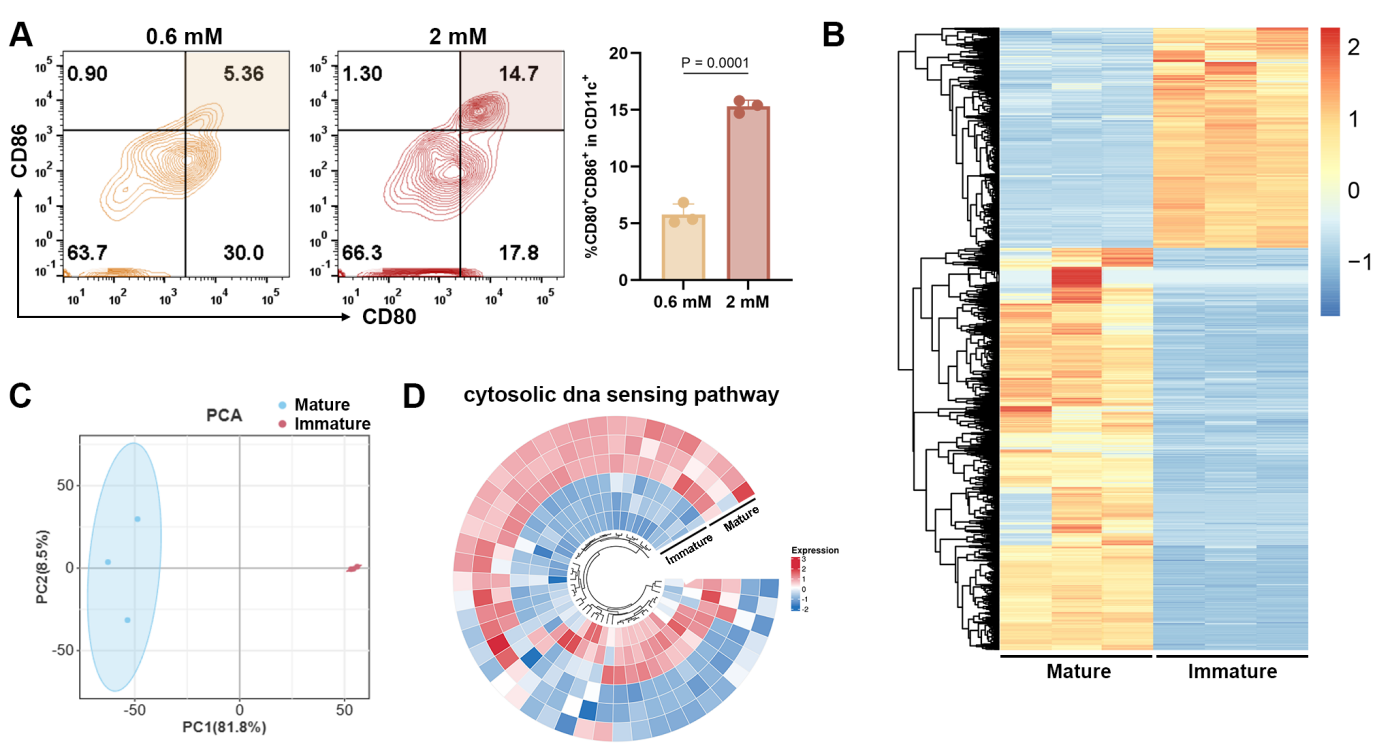


**Figure S1. Dual activation of dendritic cells by glutamine antagonists and STING agonists.** (A) Flow cytometry analysis of BMDCs treated with medium containing 2 or 0.6 mM glutamine for 24h. (B) Heatmap for differentially expressed genes in BMDCs. (C) Principal component analysis (PCA) of RNA-seq data showing distinct clustering between mature and immature BMDCs (D) Circular heatmap showing the differential expression of genes involved in the positive regulation of the cytosolic dna sensing pathway between mature and immature DCs, with data normalized by z - score. Error bars represent means ± SD. Differences between groups were tested using one-way ANOVA followed by Tukey's multiple comparisons test, or unpaired Student’s *t*-test.

**Chemical synthesis for T26**

**
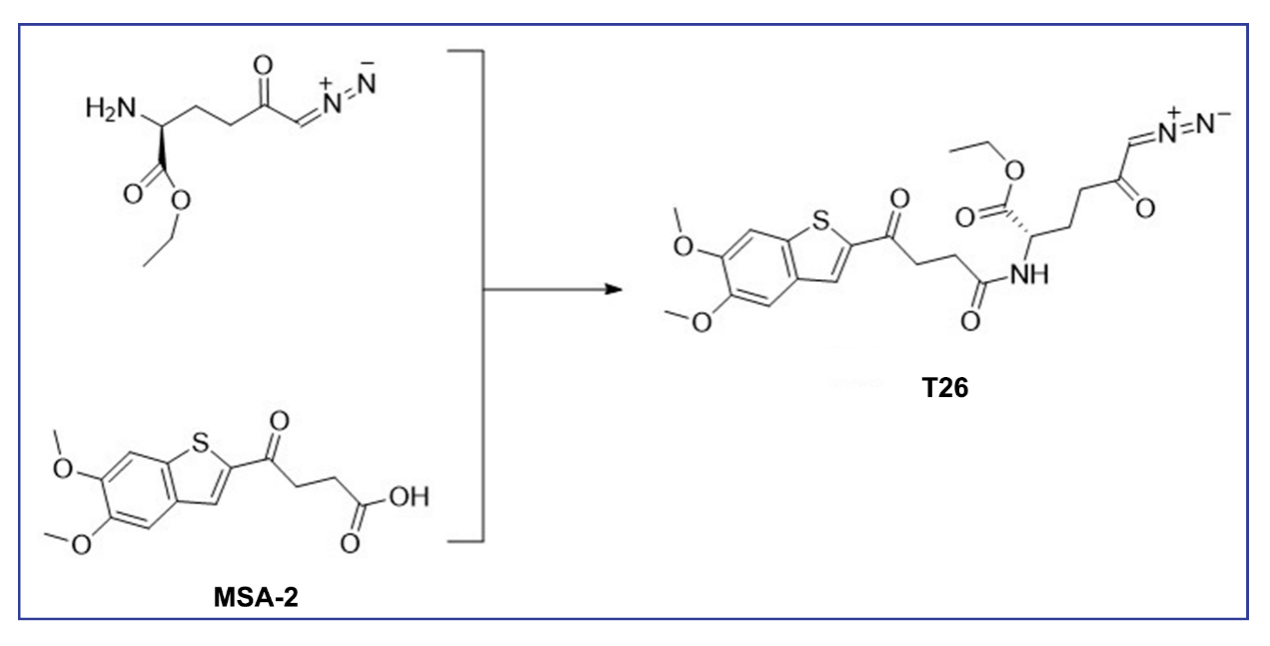
**

**Scheme S1. Synthesis routes for T26**

Synthesis for T26: MSA-2 (0.25mmol) was dissolved in 10 ml of dichloromethane (DCM) in a 50-ml round-bottom flask. Then, DIPEA (0.25mmol) and HATU (0.25mmol) were added dropwise sequentially and stirred in an ice bath for 30 minutes. Next, L-Norleucine, 6-diazo-5-oxo-ethyl ester (0.25mmol) was added, and the mixture was further stirred at room temperature for 24 hours. The completion of the reaction was monitored by thin-layer chromatography. The target products were purified by column chromatography to give a Yellow solid like substance T26 (48 mg, Yield, 41%).^1^H NMR (500 MHz, CDCl_3_) δ 7.89 (s, 1H), 7.24 (s, 2H), 6.60 (d, J = 8.0 Hz, 1H), 5.33 (s, 1H), 4.57 (td, J = 8.3, 4.6 Hz, 1H), 4.19 (qd, J = 7.1, 1.2 Hz, 2H), 3.97 (d, J = 1.4 Hz, 3H), 3.95 (d, J = 1.4 Hz, 3H), 3.47 (dt, J = 17.6, 6.9 Hz, 1H), 3.26 (dt, J = 17.7, 6.1 Hz, 1H), 2.73 – 2.62 (m, 2H), 2.50 – 2.34 (m, 2H), 2.24 (dtd, J = 14.6, 7.5, 4.8 Hz, 1H), 2.00 (dt, J = 14.5, 7.4 Hz, 1H), 1.28 (dd, J = 7.7, 6.4 Hz, 4H).^13^C NMR (126 MHz, CDCl3) δ 193.97, 192.65, 171.87, 171.79, 151.10, 148.91, 141.08, 136.80, 132.68, 129.34, 105.96, 103.56, 61.67, 56.20, 56.08, 51.86, 36.39, 33.98, 30.20, 27.41, 14.14. HRMS (ESI): calcd for [M + H]^+^ C_22_H_26_N_3_O_7_S, 476.1491; found, 476.1479. The synthetic methods of compounds 6-diazo-5-oxo-ethyl ester refer to the previous literature reports.^[1]^


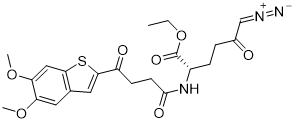

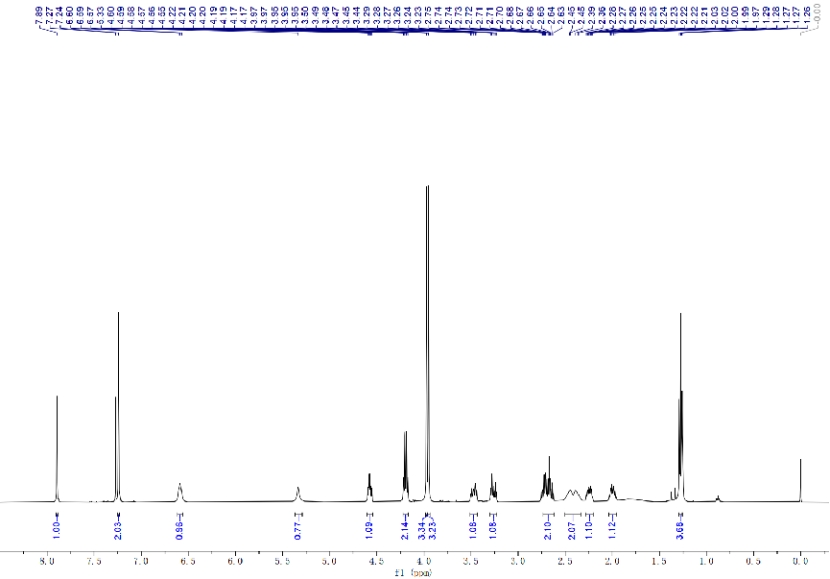


**Figure S2. ^1^H-NMR spectrum of T26**


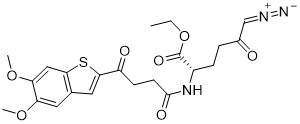

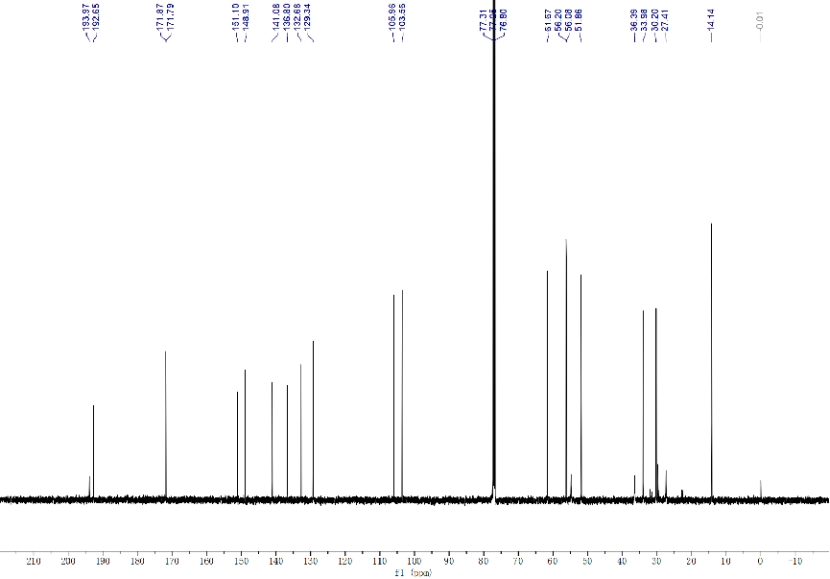


**Figure S3.^13^C-NMR spectrum of T26**


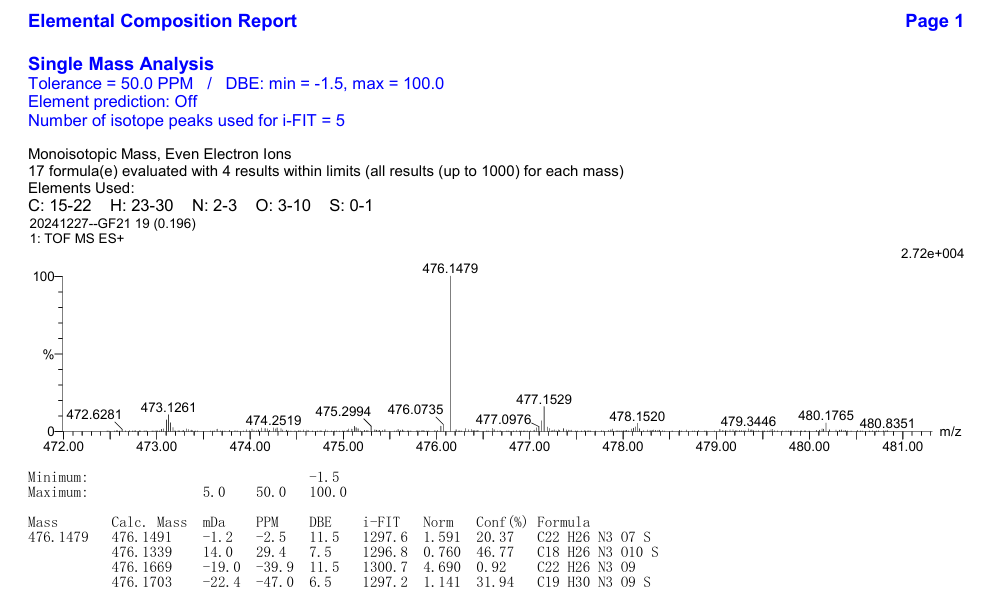


**Figure S4. HR-MS spectrum of T26.**


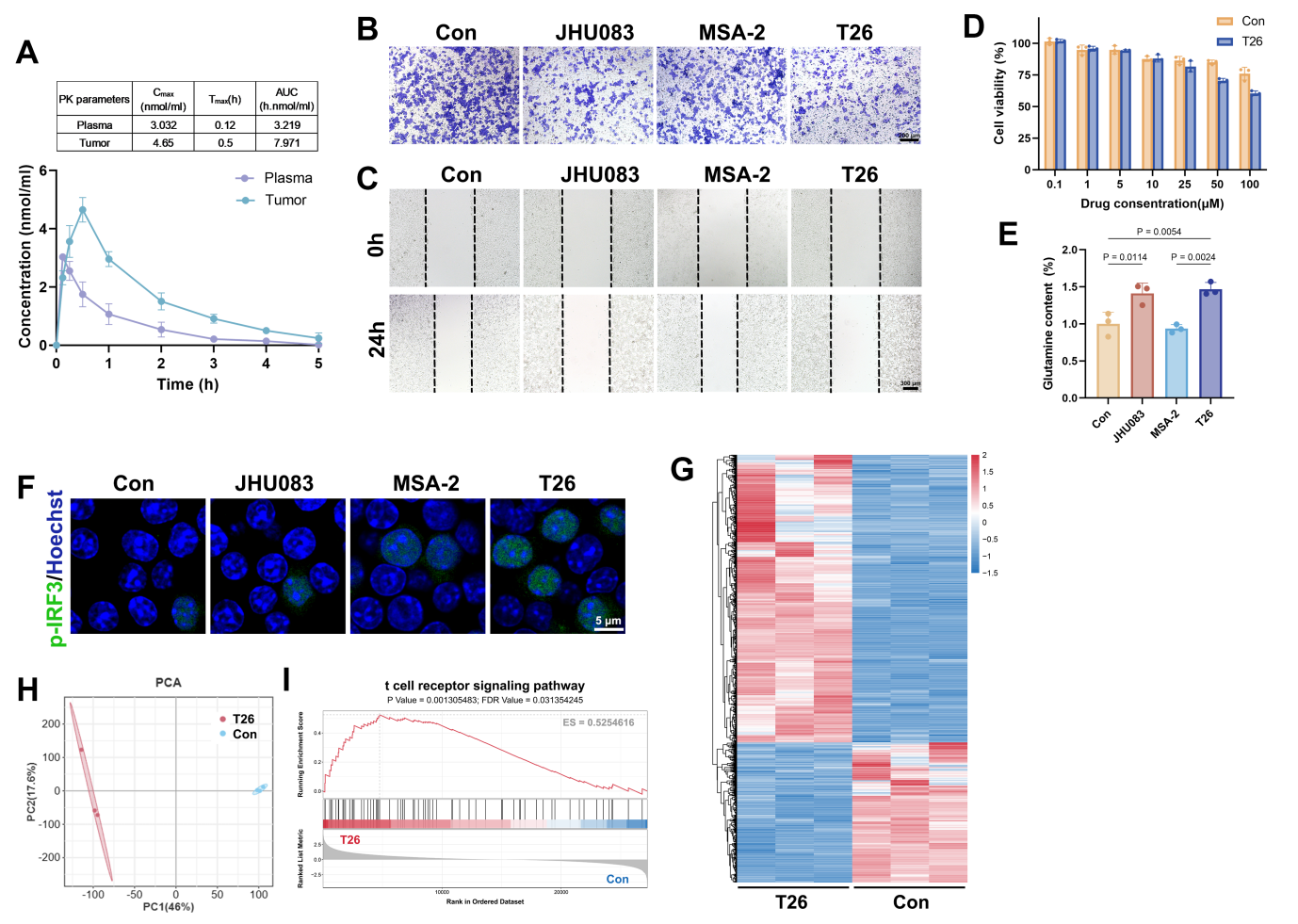


**Figure S5. Design, characterization, and functional validation of T26.**

(A) Pharmacokinetic analysis of T26 (1.5 mg/kg) in the MC38 tumor model. (B) Transwell invasion assay of MC38 cells treated with DMSO, JHU083 (5 μM), MSA-2 (5 μM) or T26 (5 μM) for 48 h. (C) Wound healing assay of MC38 cells treated with DMSO, JHU083 (5 μM), MSA-2 (5 μM) or T26 (5 μM) for 24 h. (D) MTT assay of T26 in DC2.4 cell viability. (E) Extracellular glutamine quantification in MC38 upon treated with DMSO, JHU083 (5 μM), MSA-2 (5 μM) or T26 (5 μM) for 24 h. (F) IF staining of p-IRF3 expression in MC38 cells. (G) Heatmap for differentially expressed genes in MC38, with data normalized by z - score. (H) Principal component analysis (PCA) of RNA-seq data shows distinct clustering between T26 and DMSO treated MC38. (I) GSEA showing significant upregulation of the T cell receptor signaling pathway upon T26 treatment. Error bars represent means ± SD. Differences between groups were tested using one-way ANOVA followed by Tukey's multiple comparisons test, or unpaired Student’s *t*-test.


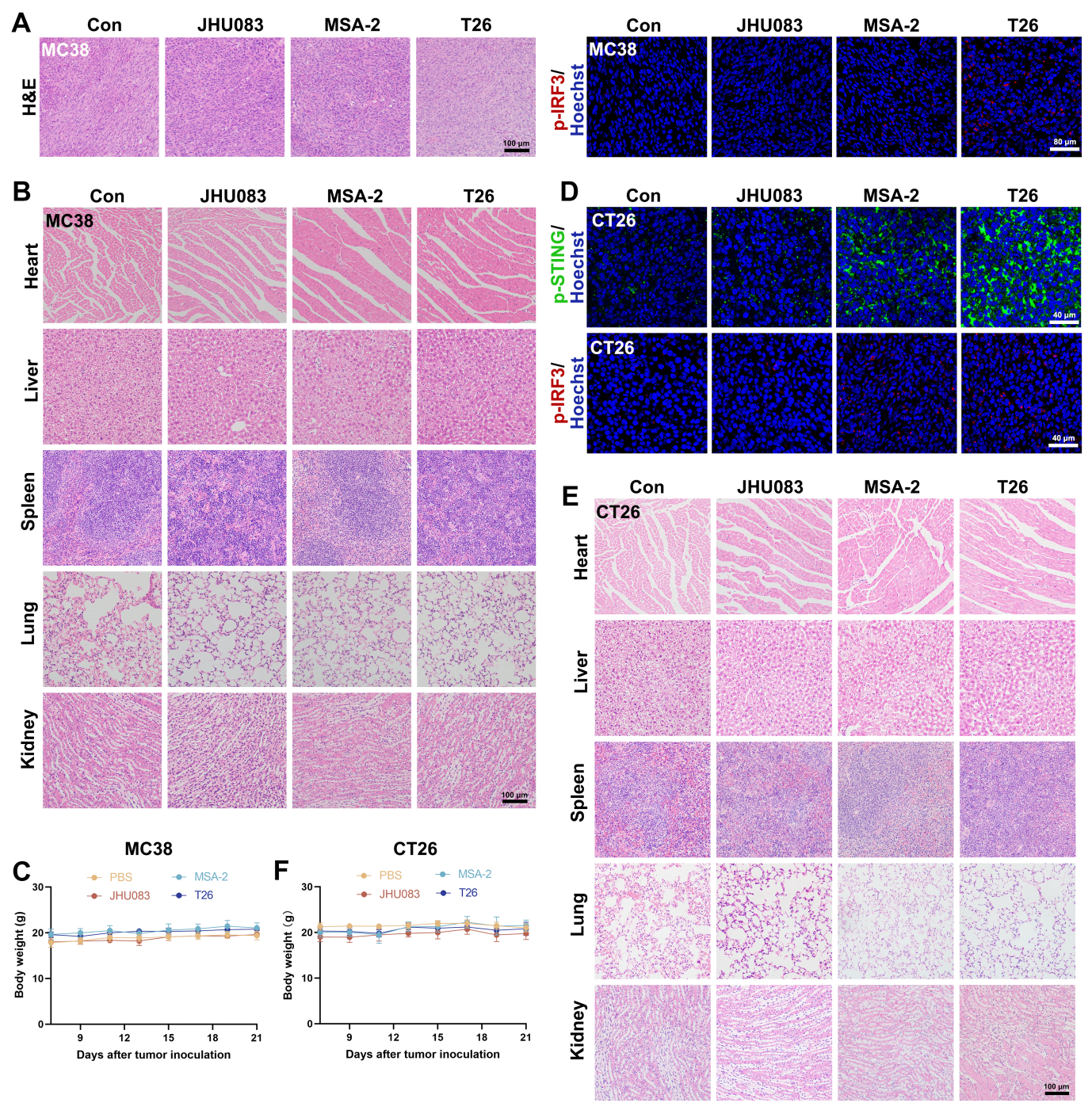


**Figure S6. Oral administration of T26 suppresses tumor growth in colorectal cancer models.**

(A) H&E staining and IF staining for p-IRF3 in MC38 tumors. (B) H&E staining of major organs from mice in the MC38 model group. (C) The body weight records of mice within the MC38 model (n = 6). (D) IF staining for p-STING and p-IRF3 in CT26 tumors. (E) H&E staining of major organs from mice in the CT26 model group. (F) The body weight records of mice within the CT26 model (n = 5). Error bars represent means ± SD. Differences between groups were tested using one-way ANOVA followed by Tukey's multiple comparisons test, or unpaired Student’s *t*-test.


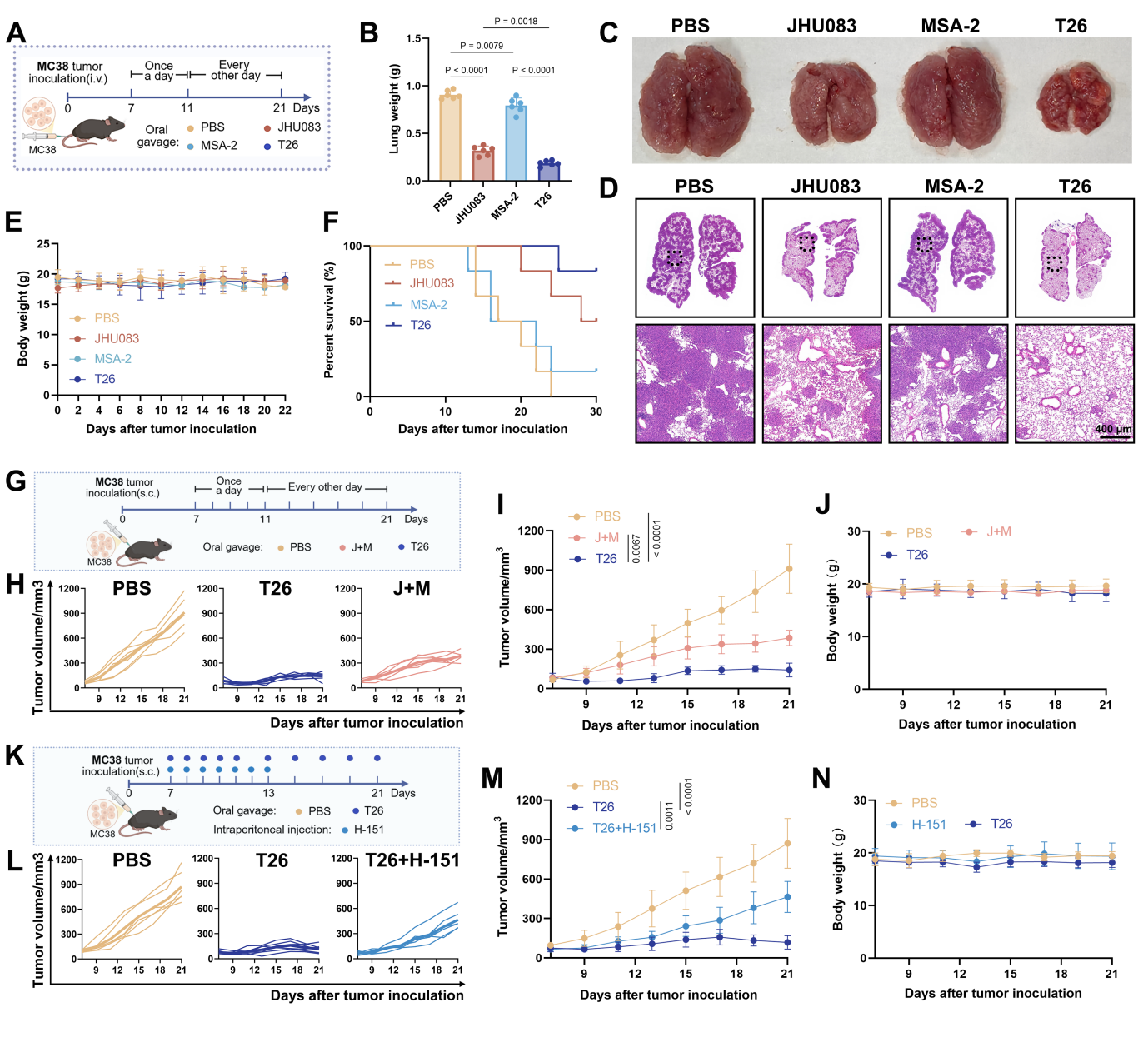


**Figure S7. Oral administration of T26 suppresses tumor growth in MC38 subcutaneous and metastatic models.**

(A) Establishment of the MC38 lung metastasis model via tail vein injection. The dosing schedule: C57BL/6 mice were inoculated with MC38 cells and treated with PBS, mice were randomly assigned to receive either JHU083 or MSA-2 at 1 mg/kg/day from day 7 to 11, followed by 0.3 mg/kg every other day from day 12 to 21, or T26 at 1.5 mg/kg/day from day 7 to 11, followed by 0.45 mg/kg every other day from day 12 to 21. (B) Lung weight assessment in the MC38 lung metastasis model (n = 6). (C) Lung images from the MC38 metastasis model after various treatments. (D) H&E staining of lung metastatic nodules in each group. (E) The body weight records of mice within the MC38 lung metastasis model (n = 6). (F) Kaplan-Meier survival analysis of MC38 lung metastasis model with different treatment (n = 6). (G) The dosing schedule: C57BL/6 mice were inoculated with MC38 cells and then randomly assigned to four treatment groups: PBS; JHU083+MSA-2 combination; T26. Mice in the combination group received JHU083 (1 mg/kg/day) and MSA-2 (1 mg/kg/day) from day 7 to 11, followed by JHU083 (0.3 mg/kg) and MSA-2 (0.3 mg/kg) every other day from day 12 to 21. The T26 group was administered T26 at 1.5 mg/kg/day from day 7 to 11, followed by 0.45 mg/kg every other day from day 12 to 21. (H-I) Tumor growth of MC38 tumors in C57BL/6 mice with different treatment. (J) The body weight records of mice within the MC38 model (n = 6). (K) The dosing schedule: C57BL/6 mice were inoculated with MC38 cells and then randomly assigned to four treatment groups: PBS control; T26; H-151+T26 combination. Mice received an intraperitoneal injection of H-151 (750 nmol in 200 μL) or PBS for seven consecutive days. The T26 and combination groups were administered T26 at 1.5 mg/kg/day from day 7 to 11, followed by 0.45 mg/kg every other day from day 12 to 21. (L-M) Tumor growth of MC38 tumors in C57BL/6 mice with different treatment. (N) The body weight records of mice within the MC38 model (n = 6). Error bars represent means ± SD. Differences between groups were tested using one-way ANOVA followed by Tukey's multiple comparisons test, or unpaired Student’s *t*-test.


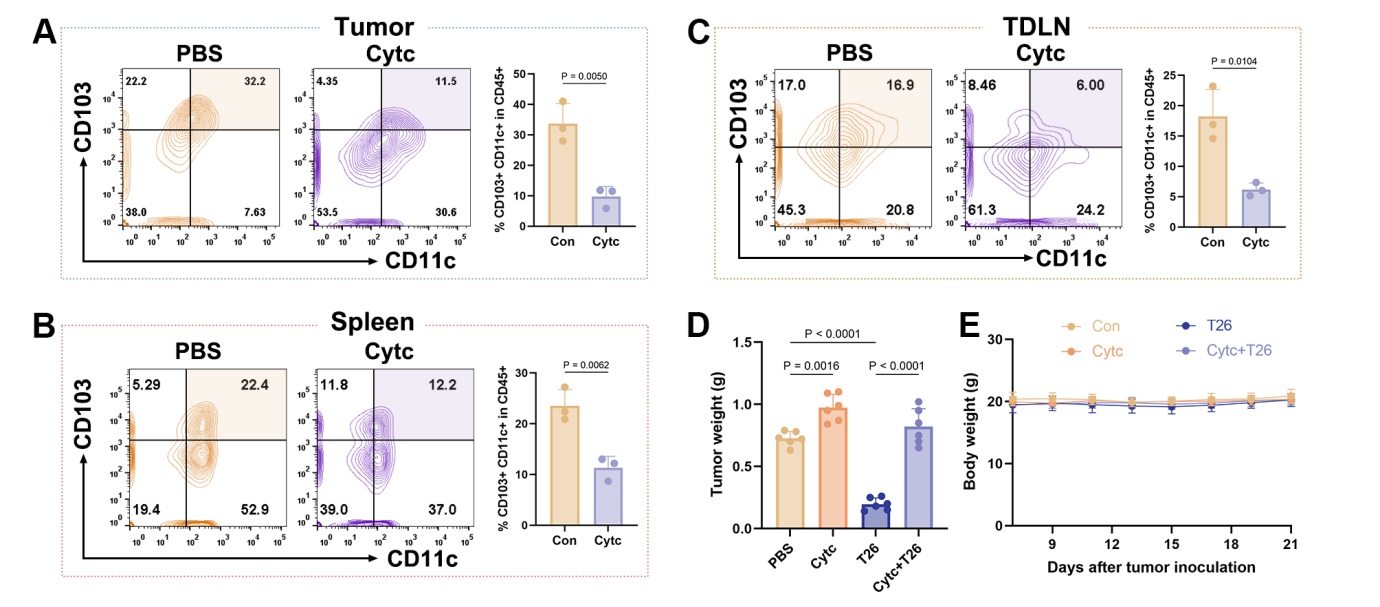


**Figure S8. T26 enhances anti-tumor immunity through DC activation.**

(A-C) Subcutaneously inject MC38 tumor cells of the C57BL/6 mice. Once the tumors became about 80 mm^3^, the mice were treated intravenously (i. v.) with Cytc vs PBS at day0, day1, day3. The blood, tumor, and TDLN were harvested at day 4 for flow cytometric analysis. The percentage of DCs (CD103⁺CD11c⁺) in tumors, spleens or TDLN of MC38-bearing mice (n = 3). (D) Tumor weight of MC38 tumors in C57BL/6 mice with different treatment. (n = 6). (E) The body weight records of mice within the MC38 model (n = 6). Error bars represent means ± SD. Differences between groups were tested using one-way ANOVA followed by Tukey's multiple comparisons test, or unpaired Student’s *t*-test.


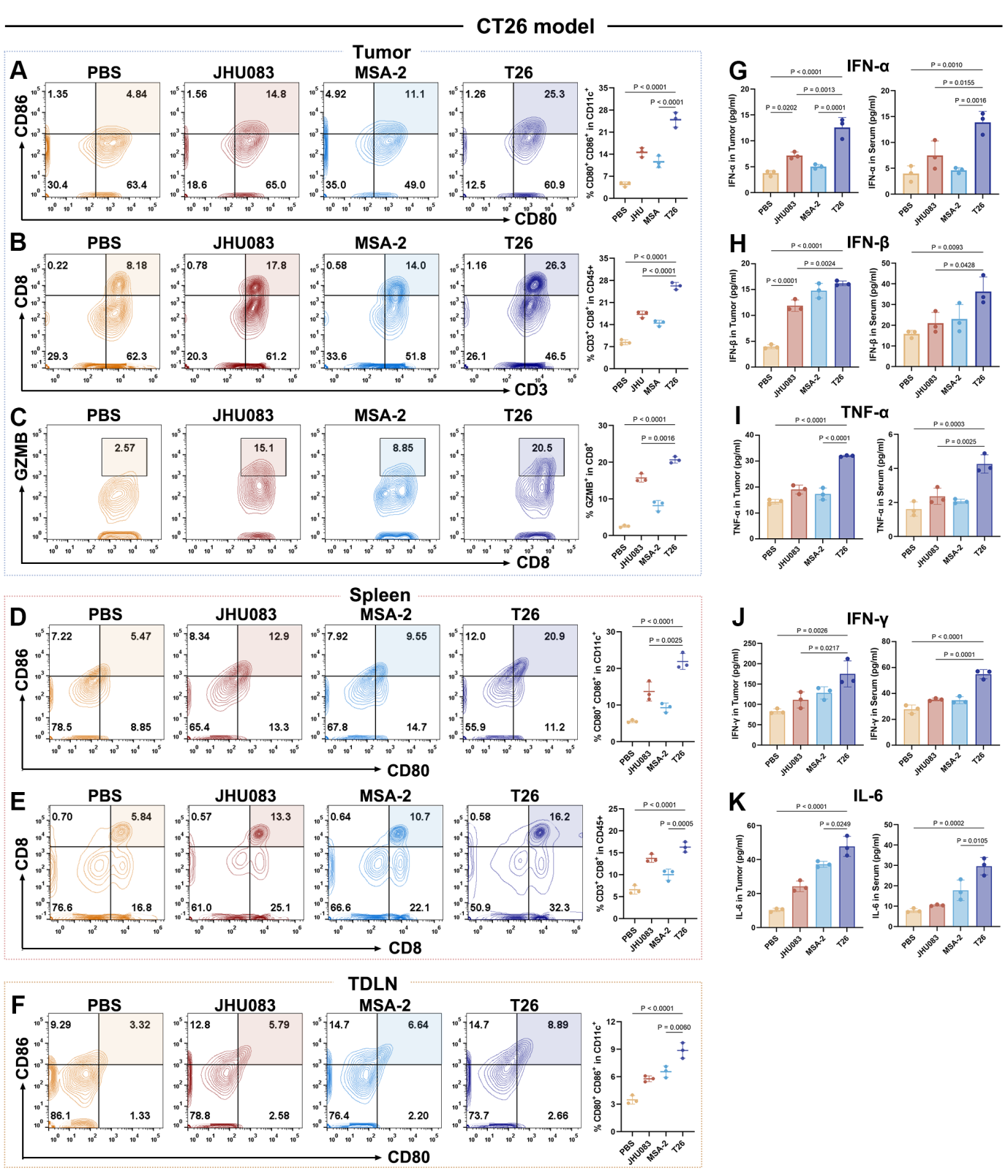


**Figure S9. T26 enhances anti-tumor immunity through DC activation in CT26-bearing mice.**

(A-C) Flow cytometry analysis of mature DCs (CD80⁺CD86⁺), CD8⁺ T cells (CD3⁺CD8⁺) and GZMB expression in tumor-infiltrating CD8⁺ T cells in CT26-bearing mice (n = 3). (D-E) Flow cytometry analysis of mature DCs (CD80⁺CD86⁺), CD8⁺ T cells (CD3⁺CD8⁺) in spleens of CT26-bearing mice (n = 3). (F) Flow cytometry analysis of mature DCs (CD80⁺CD86⁺) in TDLNs of CT26-bearing mice (n = 3). (G-K) Tumor and serum levels of IFN-α, IFN-β, IFN-γ, TNF-α, IL-6 in CT26-bearing mice (n = 3). Error bars represent means ± SD. Differences between groups were tested using one-way ANOVA followed by Tukey's multiple comparisons test, or unpaired Student’s *t*-test.


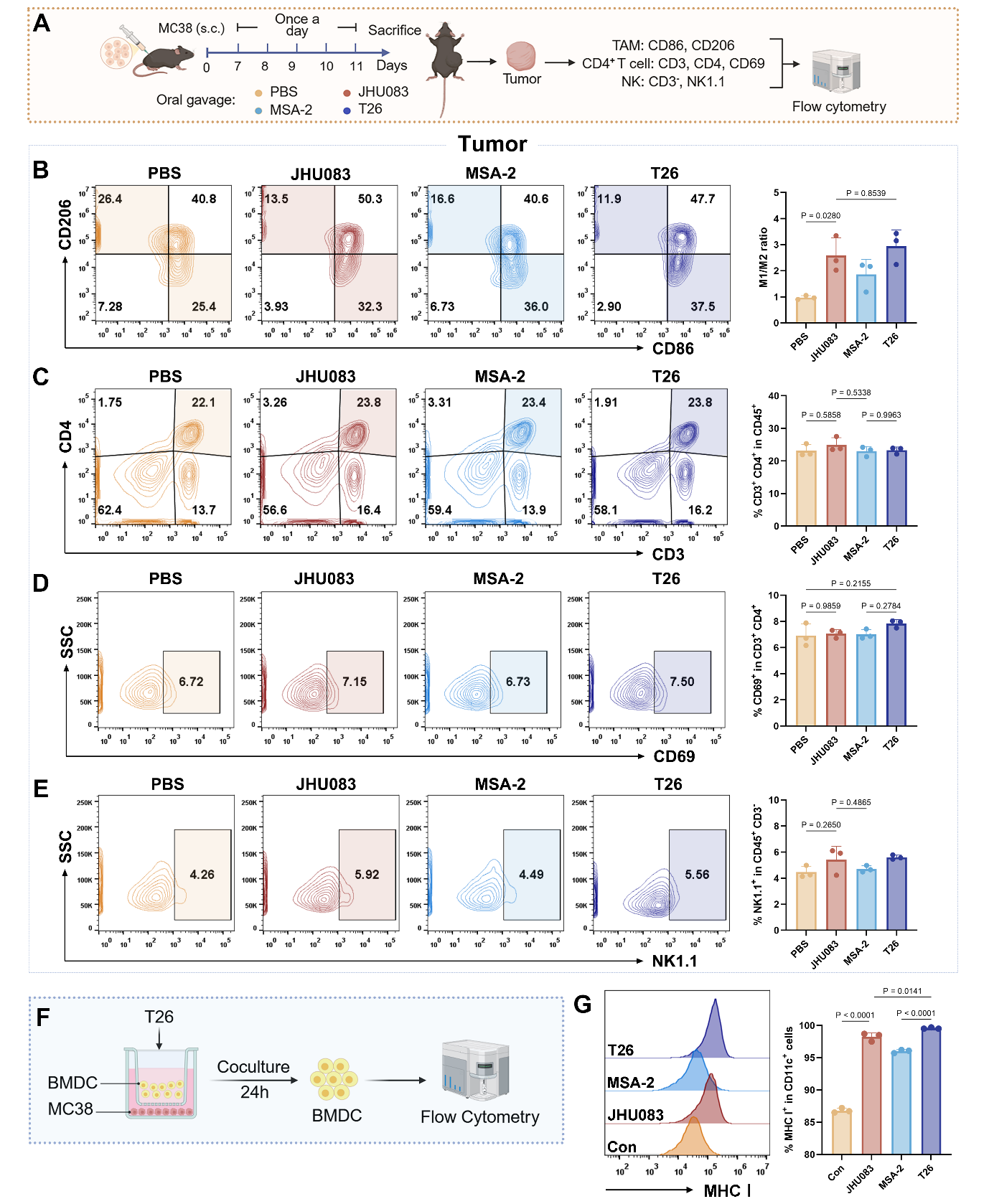


**Figure S10. T26 enhances anti-tumor immunity through specific activation of dendritic cells and CD8⁺ T cells.**

(A) Experimental workflow for analyzing immune cell populations. C57BL/6 mice were subcutaneously inoculated with 1×10^6^ MC38, into the right flank on day 0. Once tumors reached approximately 80 mm3, mice were randomly assigned to receive either JHU083 or MSA-2 at 1 mg/kg/day or T26 at 1.5 mg/kg/day from day 7 to 11. After treatment, tumors were harvested for flow cytometry analysis. (B) Polarization of tumor-associated macrophages, assessed by M1 (CD86^+^) and M2 (CD206^+^) markers in MC38-bearing mice (n = 3). (C-D) Flow cytometry analysis of CD4⁺ T cells (CD3⁺CD4⁺) and CD69 expression in CD4⁺ T cells in MC38-bearing mice (n = 3). (E) Flow cytometry analysis of NK cells (NK1.1⁺) in MC38-bearing mice. (F) Schematic of co-culture experiments of MC38 and DCs (n = 3). (G) Flow cytometry analysis of MHC I antigen presentation in BMDCs in co-culture with MC38 cells treated with DMSO, JHU083 (5 μM), MSA-2 (5 μM) or T26 (5 μM) for 24 h (n = 3). Error bars represent means ± SD. Differences between groups were tested using one-way ANOVA followed by Tukey's multiple comparisons test, or unpaired Student’s *t*-test.


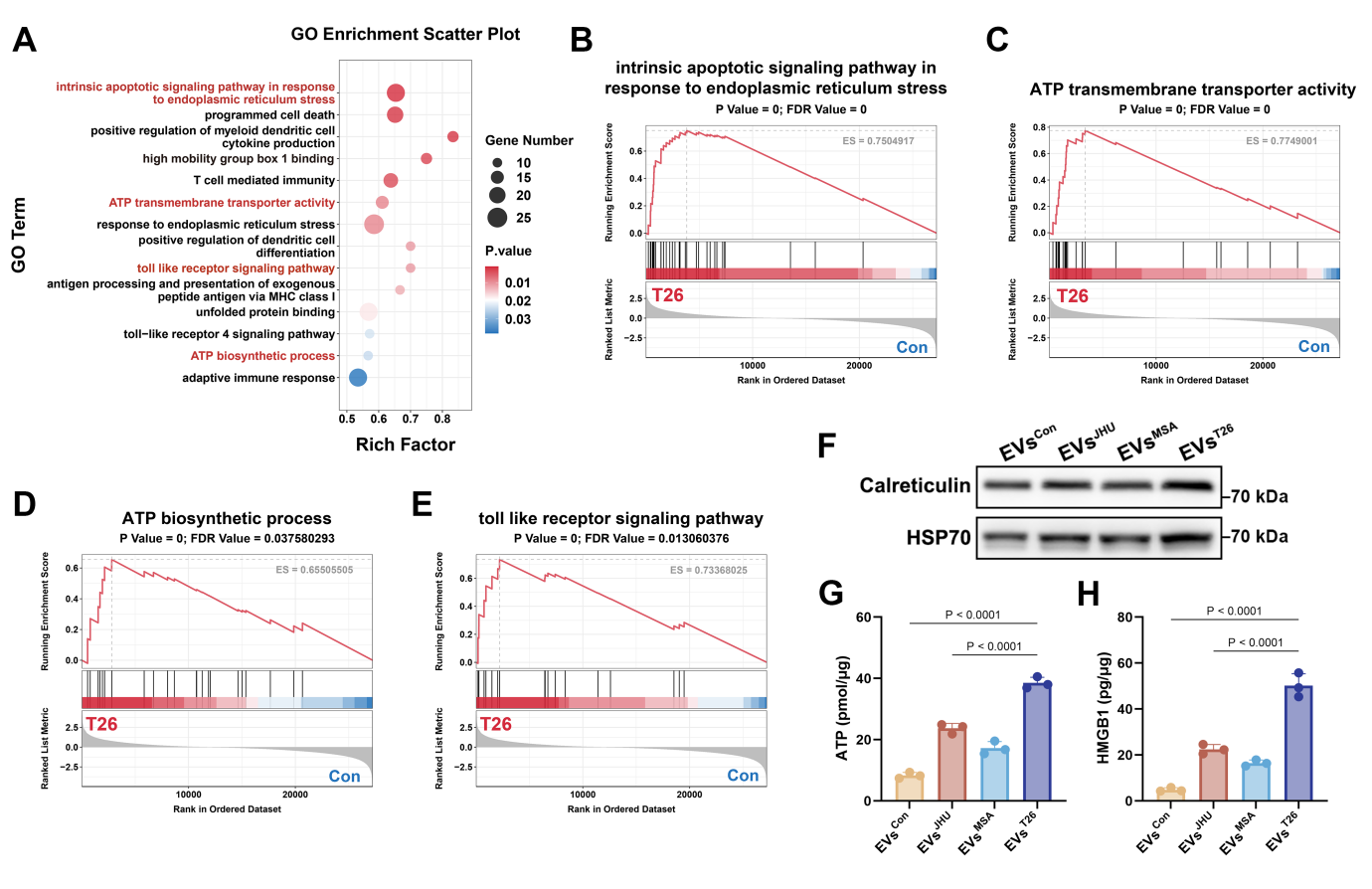


**Figure S11. T26 reprograms tumor-derived extracellular vesicles to enhance immune responses.**

(A) GO enrichment analysis of ICD-related pathways in T26-treated MC38 cells. (B-E) GSEA showing significant upregulation of the intrinsic apoptotic signaling pathway in response to endoplasmic reticulum stress pathway, ATP transmembrane transporter activity pathway, ATP biosynthetic process pathway, toll-like receptor signaling pathway upon T26 treatment. (F) Western blot analysis of EVs^Con^, EVs^JHU^, EVs^MSA^ and EVs^T26^. (G) Quantification of ATP levels of EVs^Con^, EVs^JHU^, EVs^MSA^ and EVs^T26^ (n = 3). (H) ELISA measurement of HMGB1 concentration of EVs^Con^, EVs^JHU^, EVs^MSA^ and EVs^T26^ (n = 3). Error bars represent means ± SD. Differences between groups were tested using one-way ANOVA followed by Tukey's multiple comparisons test, or unpaired Student’s *t*-test.


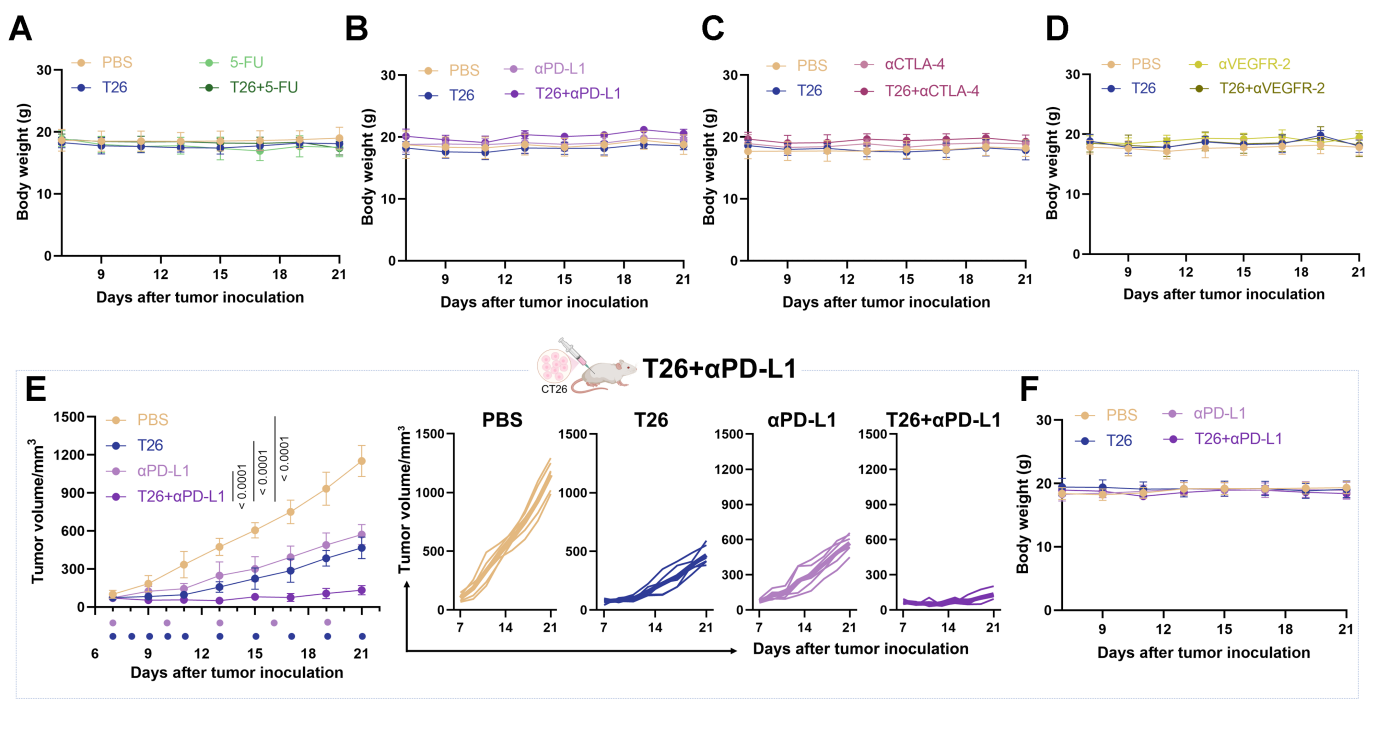


**Figure S12. Synergistic tumor suppression by T26 in combination therapies.**

(A) The body weight changes of MC38 tumor-bearing mice that received the combination therapy of T26 and 5-FU. (B) The body weight changes of MC38 tumor-bearing mice (n = 6) that received the combination therapy of T26 and αPD-L1. (C) The changes in body weight of MC38 tumor-bearing mice (n = 6) when administered the combined treatment of T26 and αCTLA-4. (D) The body weight changes of MC38 tumor-bearing mice that received the combination therapy of T26 and αVEGFR-2 (n = 6). (E) Combined treatment with T26 (1 mg/kg/day from day 7 to 11, followed by 0.3 mg/kg every other day from day 12 to 21, intragastric gavage) and αPD-L1 (2 mg/kg, intraperitoneal injection) in CT26 tumor-bearing mice. Synergistic efficacy and tumor growth curves of the αPD-L1 + T26 group were compared with those of the PBS-treated, T26-treated, and αPD-L1-treated groups (n = 6). (F) Kaplan-Meier survival analysis of CT26-bearing mice with different treatment (n = 6). Error bars represent means ± SD. Differences between groups were tested using one-way ANOVA followed by Tukey's multiple comparisons test, or unpaired Student’s *t*-test.

**Reference**

[1] R. Rais, A. Jančařík, L. Tenora, et al., *“Discovery of 6-Diazo-5-oxo-l-norleucine (DON) Prodrugs with Enhanced CSF Delivery in Monkeys: A Potential Treatment for Glioblastoma,”* *The Journal of Medicinal Chemistry* **2016**, 59 (18), 8621.

doi:10.1021/acs.jmedchem.6b01069
